# Large depth-of-field ultra-compact microscope by progressive optimization and deep learning

**DOI:** 10.1038/s41467-023-39860-0

**Published:** 2023-07-11

**Authors:** Yuanlong Zhang, Xiaofei Song, Jiachen Xie, Jing Hu, Jiawei Chen, Xiang Li, Haiyu Zhang, Qiqun Zhou, Lekang Yuan, Chui Kong, Yibing Shen, Jiamin Wu, Lu Fang, Qionghai Dai

**Affiliations:** 1 Department of Automation, Tsinghua University, 100084 Beijing, China; 2 Institute for Brain and Cognitive Sciences, Tsinghua University, 100084 Beijing, China; 3 Beijing Key Laboratory of Multi-dimension & Multi-scale Computational Photography (MMCP), Tsinghua University, 100084 Beijing, China; 4 Beijing Laboratory of Brain and Cognitive Intelligence, Beijing Municipal Education Commission, 100084 Beijing, China; 5 Tsinghua Shenzhen International Graduate School, Tsinghua University, 518055 Shenzhen, China; 6 State Key Laboratory of Modern Optical Instrumentation, Zhejiang University, 310027 Hangzhou, China; 7 OPPO Research Institute, 518101 Shenzhen, China; 8 Tsinghua-Berkeley Shenzhen Institute, Tsinghua University, 518055 Shenzhen, China; 9 School of Information Science and Technology, Fudan University, 200433 Shanghai, China; 10 Department of Electronic Engineering, Tsinghua University, 100084 Beijing, China

**Keywords:** Optical imaging, Microscopy, Machine learning

## Abstract

The optical microscope is customarily an instrument of substantial size and expense but limited performance. Here we report an integrated microscope that achieves optical performance beyond a commercial microscope with a 5×, NA 0.1 objective but only at 0.15 cm^3^ and 0.5 g, whose size is five orders of magnitude smaller than that of a conventional microscope. To achieve this, a progressive optimization pipeline is proposed which systematically optimizes both aspherical lenses and diffractive optical elements with over 30 times memory reduction compared to the end-to-end optimization. By designing a simulation-supervision deep neural network for spatially varying deconvolution during optical design, we accomplish over 10 times improvement in the depth-of-field compared to traditional microscopes with great generalization in a wide variety of samples. To show the unique advantages, the integrated microscope is equipped in a cell phone without any accessories for the application of portable diagnostics. We believe our method provides a new framework for the design of miniaturized high-performance imaging systems by integrating aspherical optics, computational optics, and deep learning.

## Abstract (web summary)

Traditional optical microscope, while bulky, often fails to deliver optimal performance. Here, the authors have engineered an integrated microscope of 0.15 cm^3^ in volume and a weight of 0.5 g, which outperforms a commercial microscope and can be seamlessly integrated with a smartphone.

## Introduction

Microscopy is an indispensable tool in understanding the world that cannot be seen with the unaided eye and facilitates diverse applications in fundamental biology^1^, systems neuroscience^2^, and clinical diagnostic^3^. Most of the microscopes require tabletop optical instrumentations, including multiple glass lenses and bulky sensors, as well as trained personnel for operations. However, the complexity prevents accessibility in resource-limited settings and hampers the scope and scale of applications. Even with that bulkiness, the development of the microscope is confounded in several aspects. Scale-dependent geometric aberrations limit the resolution of the microscope in the margin of a millimeter-scale field-of-view (FOV), resulting in an undesirable trade-off between the effective space-bandwidth product (SBP) and the complexity of the optical design^4^. High resolution is always desired in microscopic systems, but the depth-of-field (DOF) is inevitably reduced due to the high numerical aperture (NA) and leads to poor imaging quality for 3D distributed samples^5^. Emergent advances in sophisticated optical design try to circumvent these restrictions through complex lens configuration^6^ and multi-view information acquisition^7,8^, which achieve remarkable results in table-level laboratory equipment, but the bulkiness is even more problematic.

Miniaturized integration is a pivotal advance that facilitates low-cost production and typically leads to improved performance and broad applications in telecommunications, computing, and genomics^9^. Recently, a miniaturized microscope has achieved breakthroughs in multiple aspects, including neural recording in freely behaving mouse^9,10^, high-throughput screening^11,12^, and flow cytometry^13,14^. Further with computational enhancement, the extended DOF (EDOF) that provides robustness over rough surfaces of 3D samples^15^ can be achieved together with corrected color and enhanced resolution^16–18^. However, the optical performance of current miniaturized microscopes is still limited in size, performance, and cost. Approaches with simple lenses are limited in sub-millimeter FOVs with remarkable distortions^19–21^, while larger FOVs can be achieved through more complex lens combinations, but the overall length and weight of the system increase rapidly^22,23^. Although two-photon and three-photon-based miniaturized microscopes have been developed to provide deep penetration with optical sectioning^24–26^, they require more specialized optical elements and suffer from low acquisition speed for high-throughput imaging. Moreover, limited space for placing multiple compound lenses makes most miniaturized microscopes monochromatic^9,27,28^. Integrated light microscope designs that break those limitations remain to be explored.

Recently, deep optics technologies that parallel optimize the optical design and image processing algorithms are emerged and promising to achieve superior performance^29,30^ than traditional ray-tracing-based optical designs. The end-to-end fashion in deep optics has been validated to be distinguished in achieving large FOV^31,32^, large DOF^33^, high dynamic range^34^, and hyperspectral imaging^16^, among others. However, current deep optics techniques have been limited in simplistic optical systems and remained a great challenge for applications with small working distances and large FOVs due to the ever-larger solution spaces and aberrations in microscopic applications^29^. In addition, most deep neural networks for megapixel-level microscopic image restoration require large storage spaces and computational resources, which are hard to be distributed in integrated systems for practical use.

To overcome these limitations, we develop a progressive optimization pipeline to exploit state-of-the-art optical design techniques in computational imaging systems, together with physics-based deep-learning reconstructions compositely. Specifically, the progressive optimization paradigm first constrains the heavily non-linear and complicated design space into a feasible size by ray-tracing-based merits and leverages advanced artificial intelligence algorithms to exhaustively rummage the optima, with over 30 times memory reduction compared to the end-to-end optimization paradigm. We consequently build a compact multi-color microscope that is as light as 0.5 g in a 0.15 cm^3^ volume and can even be integrated into a cell phone for potable diagnosis. Inspired by emerging technologies in diffractive elements^35^, we integrate a cubic phase mask to achieve an EDOF of 300 µm for 0.16 NA acquisition that is tenfolds of the commercial system and in the single-dollar range for mass production. With four aspherical lenses optimized to generate spatially uniform coded point spread functions (PSF), our device achieves 3 µm optical resolution across a wide FOV with a diameter larger than 3.6 mm after learning-based reconstructions. A physics-aware model is established to simulate the forward imaging process of the integrated microscope, which can fuel the training of the recovery algorithm to accomplish ground-truth-deficient restoration and perpetuate the generalization ability. We further apply a pruned deep neural network as the image recovery module, offering the powerful capability of resolving high-fidelity information in a noniterative, feed-forward manner, but with near 80% parameter reductions for real-time processing of megapixel level captures, which is critical for ready distributions in mobile platforms. Thereby, not only compressing over 100,000 times in volume, our integrated microscope obtains imaging performance beyond a commercial 5× microscope with over 10 times improved DOF, which is necessary for practical applications on rough surfaces of most samples across a large FOV. Even compared with existing advanced miniaturized microsopes^27,36–40^, the proposed integrated microscope has a much smaller size and weight (Supplementary Table 1). In the meantime, the total cost of the system is below 10 dollars for mass production with the plastic lenses used and no cemented lenses involved. To demonstrate its unique advantages, we used the microscope for mobile health monitoring after integration into a commercial cell phone. By detecting skin moisture with over 80% accuracy, we show the great potential of the proposed integrated microscope in image-based diagnostics and high-throughput screening on a generally accessible mobile platform. We further open source the design of the proposed integrated microscope (Supplementary Table 2) and corresponding restoration network (Code Availability and additional online data^41^) and hope they spur the development of high-performance integrated optical devices.

## Results

### High-performance integrated system by progressive optimization

To accomplish high-quality imaging in an integration platform with minimized size and maximized depth of field (DOF), pivotal challenges from geometrical aberrations, resolution, and DOF dilemma, and chromatic aberrations are necessary to be remedied. First, the effective space-bandwidth product of an optical system reduces rapidly with the reduction of the lens scale due to the practical limit set by the geometrical aberrations^42^. Second, intrinsic tradeoffs between the spatial resolution and DOF impair the performance either in capturing delicate structures or in being robust over rough 3D samples. Third, chromatic aberrations in miniaturized devices are raised as diffractive elements with complex surfaces used and hamper wide applications of multi-channel screening and color-coded neural imaging.

**Fig. 1:**
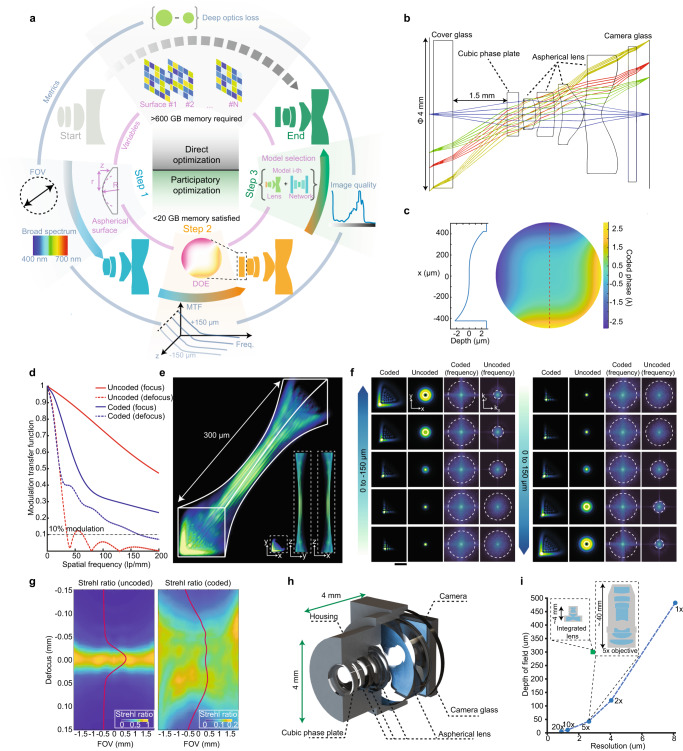
Principles of progressive optimization of an integrated microscope. **a** Progressive optimization pipeline for a high-performance integrated microscope. In the first step, the integrated microscope consisting of plastic lenses is optimized with field of view (FOV) and chromatic merits through a canonical ray-tracing approach. In the second step, a diffractive optical element (DOE) containing cubic phase distribution is inserted in the front of the integrated microscope, and the system is further optimized for consistent modulation transfer function (MTF) across a 300-µm depth range. For every amplitude of the cubic phase (“Methods”), a corresponding lens system is optimized, and multiple candidate models are formed. In the third step, we separately train a deep neural network to retrieve clear images from captures of each of the models, and select the one yielding the best quality as the final optical design. As a comparison, directly optimizing all surfaces with deep optics algorithms needs 16 million calculation grids in an aperture of 4 mm and a feature size of 1 µm for each surface, consuming over 600 GB of memory. On the other hand, our progressive but systematic optimization can be finished in a desktop-level computer with 20 GB of memory. **b** Wireframe sketches of aspherical surfaces and DOE (here is a cubic phase plate) in the proposed integrated microscope. Irregular surfaces in our integrated microscope help achieving superior performance compared to spherical surfaces (Supplementary Fig. 1). **c** Modulation phase of the DOE in the integrated microscope (right) and corresponding surface fluctuations across the red dashed line (left). The valid area of DOE is lower than the surface of the surrounding glass to protect the component. **d** MTF characteristics of coded (with DOE) and uncoded (without DOE) PSFs in the focal depth (*z* = 0 µm) and defocused depth (*z* = 150 µm). **e** 3D PSF in the center of FOV across 300 µm depth. The maximum intensity projection (MIP) from *x*, *y*, and *z* axis are plotted below. **f** Spatial and frequency plots of the PSF without DOE and with DOE through different depths. White circles show a valid frequency range. **g** Strehl ratio across different FOV and depth of field (DOF) of uncoded microscope (without DOE, left) and coded microscope (with DOE, right). Red solid lines in each panel present the normalized FOV-averaged Strehl ratios. The colormap range has been adjusted for better visibility. **h** 3D rendering of the proposed integrated microscope. **i** The DOF changes with optical resolution of commercial 1×, 2×, 5×, 10×, and 20× objectives (blue). The proposed integrated microscope achieves high resolution that is comparable to the commercial 5× objectives but with 10 times increased DOF (green) and orders of magnitude smaller size.

To solve all these problems, we propose an advanced progressive design pipeline that fully leverages the advantages of both traditional ray-tracing-based and emerging deep-optics-based optimizations compositely (Fig. 1a). We notice that direct optimization of both optical system and retrieving algorithms in an end-to-end manner requires over 16 million calculation grids per surface and 600 GB memory consumption (Supplementary Note 3), inevitably leads to suboptimal solutions with huge computational costs. Instead, we first narrow down the overall design space with traditional optical design merits to achieve an integrated lens design with fair performance and compact size (Fig. 1b), then jointly optimize both lenses and a coding phase plate for DOF extension, and finally concurrently optimize the overall system with a neural network to achieve the best performance across all DOF.

In the first round of optimization, our integrated system accomplishes an optical resolution of 3 µm across a FOV of Φ3.6 mm and in a conjugate distance of 6 mm with four pieces of aspherical lens under traditional ray-tracing-based merit. To achieve this, we use a multi-dimensional coupling optimization design to realize equivalized MTF across a wide wavelength range (470–650 nm, step 1 in Fig. 1a). We adopt two kinds of optical plastic materials as well as aspherical surface shapes to effectively reduce the chromatic aberrations while keeping the form factor compact without using cemented doublets^43^ (“Methods”). The strict Rayleigh-Sommerfeld diffraction theory is used to establish the corresponding optical propagation model, and adaptive gradient descent is applied to optimize the surface shape to reduce aberrations. As a comparison, a conventional microscope system that is consisted of spherical glass lenses (Supplementary Fig. 1) uses six more lenses (including two sets of cemented doublets to eliminate chromatic aberration), and its conjugate distance (11.735 mm) is twice that of the proposed system. In the second round of optimization, we introduce a diffractive optical element (DOE) featuring a cubic phase distribution in close proximity to the lens system’s pupil plane, facilitating light field encoding and bolstering the depth invariance of the point spread function (PSF) (Fig. 1c). As a member of the polynomial-based asymmetric phase profile family, the cubic phase is capable of producing MTFs that demonstrate gradual variation in response to focus shift, thereby extending the DOF effectively^44^. For a specific cubic phase parameter, we finetune the surface parameters of the integration system such that MTFs of the system are similar across 300 µm (“Methods”, Fig. 1d, step 2 in Fig. 1a). The optimized PSFs then show similar triangle profiles across 300 µm depth range (Fig. 1e) with a nearly unchanged frequency modulation range (Fig. 1f), while the uncoded PSFs quickly lose modulations in the higher frequency range during defocus. We then get 15 configurations with varied cubic phase parameters and lens shapes, and we separately train a neural network for each configuration with merits of best reconstructions across the 300-µm depth range (step 3 in Fig. 1a). We choose the configuration with the best imaging quality as our final design (Supplementary Fig. 12).

After the optimization, the proposed integrated microscope shows non-degraded Strehl ratios across a 300-µm depth range and Φ3.6 mm lateral FOV, which is a widely used merit that evaluates the perfectness of an optical system. On the other hand, the system without the DOE coding shows substantially lower Strehl ratio when defocus is beyond 30 µm (Fig. 1g). The size of the optimized lens system is smaller than 4 mm in all dimensions without the sensor board (Fig. 1h). Noteworthily, the optical resolution and FOV of the progressively optimized integrated microscope are comparable to a commercial microscope with a 5× objective (Fig. 1i), while the overall volume is reduced by 5 orders of magnitude (“Methods”). The corresponding full design characteristics can be found in Supplementary Fig. 2.

### Deep-learning-enhanced image restoration in the integrated microscope

**Fig. 2:**
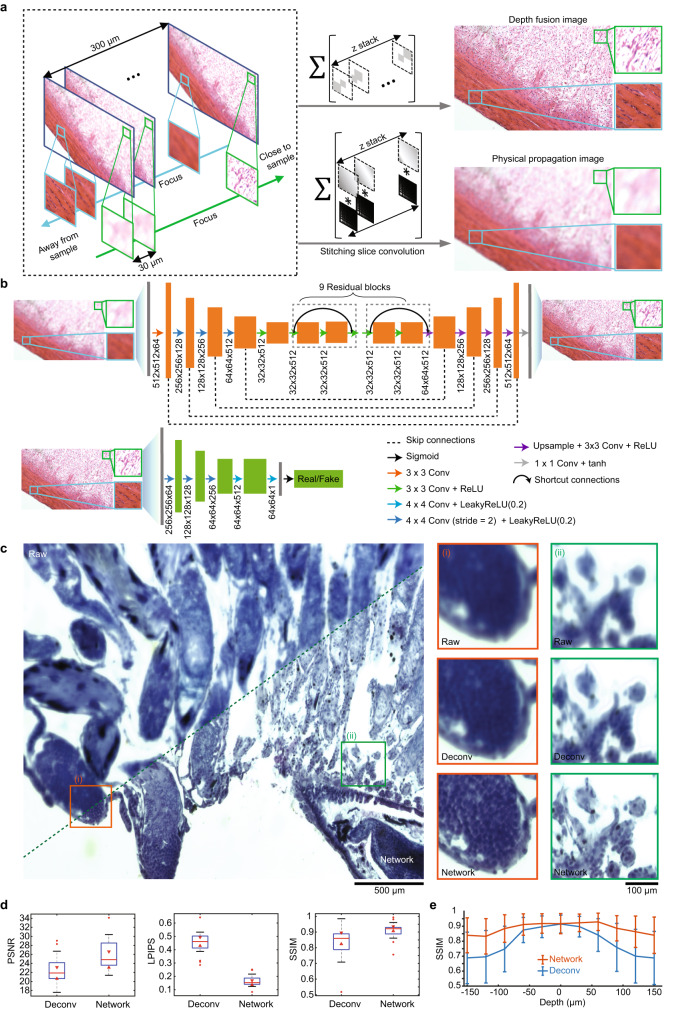
Simulation-supervised deep neural network for the integrated microscope. **a** Illustrations of generating training pairs through simulation-supervision strategy. To generate the training pairs between an all-in-focus image and a depth-coded image from the integrated microscope, a commercial microscope combined with a piezo objective scanner was employed to capture focal stacks of 3D samples (Supplementary Fig. 13). Two regions from a tilted sample with an approximate axial distance of 300 µm were delineated by blue and green boxes. The region labeled by the green box was situated in the top-right corner of the image and gradually came to focus as the imaging focal plane approached the sample. Conversely, the region marked by the blue box was situated in the bottom left corner of the image and gradually progressively became clearer as the imaging focal plane receded from the sample. The green and blue arrows represented the direction in which the focal plane must move to capture those regions with optimal clarity. The Σ symbol denoted the summation of a collection of images enclosed within a large bracket. In the “Depth fusion” row, each slice from the captured focal stack was first processed to extract the region where the sample was clearly captured (i.e., the in-focus region), as illustrated by gray patches inside the bracket. Then, these in-focus regions were summed to create an all-in-focus image. In the “Physical propagation image” row, each slice from the captured focal stack was first convolved with the depth-specific PSFs and then summed to reassemble the capture from the integrated microscope. **b** Structures of the proposed simulation-supervision network to retrieve clear images from the coded integrated microscope. **c** Comparisons of the raw coded image (Raw; top left) and the network-retrieved image (Network; bottom right). The zoom-in regions on the right side compare the raw coded images (Raw; top), deconvolved images using shift-variant deconvolution (Deconv; middle), and network-retrieved images (Network; bottom). Representative data from 122 samples. **d** Statistical comparisons between the shift-variant deconvolution and the proposed network on 19 test samples in terms of peak signal-to-noise ratio (PSNR, left), perceptual loss (Learned Perceptual Image Patch Similarity, LPIPS^70^, middle), and structure similarity index (SSIM, right). Central line inside the box: Median. Box: interquartile range. Whiskers: Maximum and minimum. Outliers: Individual data points. **e** Statistical comparisons between results of the shift-variant deconvolution (blue) and the proposed network (red) for 19 samples placed at different axial depths in terms of SSIM. Error bars are represented for standard deviation. Center of error bars: Mean of scores.

Deep learning is a powerful technique that performs complex operations using a multilayered artificial neural network and has shown great success in various computer-vision tasks^45^. We incorporate a deep neural network in the final optimization stage and envisage high-quality reconstructions from DOE-coded raw images in both the training and practical usage stage. However, it is hard to capture the paired ground-truth images that remain clear and sharp in different depths in practice, ensuing the restoration problem of ground-truth deficiency^46^. We thus accomplish the image restoration task through the simulation-supervision approach. We first use a standard 5× tabletop microscope to capture images at different depths with a motorized stage (Fig. 2a, “Methods”) and then digitally propagate the defocused images through the proposed integrated microscope and simulate corresponding blurry captures (“Methods”, Supplementary Figs. 3, 13). Combining coded images that are defocused differently thereby generates the input of the network. On the other hand, we utilized image fusion technology^47^ to stitch clear parts in each defocused image to form the all-in-focus ground truth across the DOF. The virtually generated input and label form training pairs to fuel the neural network in an end-to-end manner to restore clear images from blurred captures (Fig. 2b, “Methods”, Supplementary Software 1). By separately optimizing the network for each optical setup in the proposed progressive optimization, we simultaneously achieve both the optimized reconstruction algorithms and optical models, consolidating the superior performance of the proposed integrated microscope.

The effectiveness of the above simulation-supervision framework working on practical data is assured through highly similar distributions of features from simulated coded captures and real coded captures (Supplementary Fig. 4). After proper training, the neural network can efficiently remove blurriness from experimentally coded captures (Fig. 2c, Supplementary Fig. 5). To show the superior restoration ability of the proposed simulation-supervision network, we compare it with the state-of-the-art shift-variant deconvolution algorithms which have shown great success with irregular nonuniform PSFs across a large FOV^37,48^ (Supplementary Fig. 6). We find that our simulation-supervision method outperforms that technique in terms of peak signal-to-noise ratio (PSNR), perceptual loss^49^, and structure similarity index^50^ (SSIM; Fig. 2d). We further quantitatively measure the fidelity of retrieved images across multiple depths (Fig. 2e). The quality scores by the proposed neural network maintain similar high performance across 300-µm depth ranges, while scores of the shift-variant deconvolution algorithm quickly degrades when the defocus is beyond 50 µm. Compared to refocusing methods that directly retrieve large DOF images without DOE coding^51^, our simulation-supervision method also achieves superior results (Supplementary Fig. 7).

Recent emerging unsupervised learning technology establishes network mappings between domains without paired data^52^. Although the unsupervised manner avoids the procedure of focal stack acquisition compared to our simulation-supervision approach, we find that our approach achieves better performance in PSNR, SSIM, and perceptual loss (Supplementary Fig. 8). Besides, the unsupervised approach generates many artifacts in the boundary of features (Supplementary Fig. 8a, b), while our simulation-supervision approach achieves vivid reconstructions without artifacts.

### Evaluation of the mass-producible integrated microscope

**Fig. 3:**
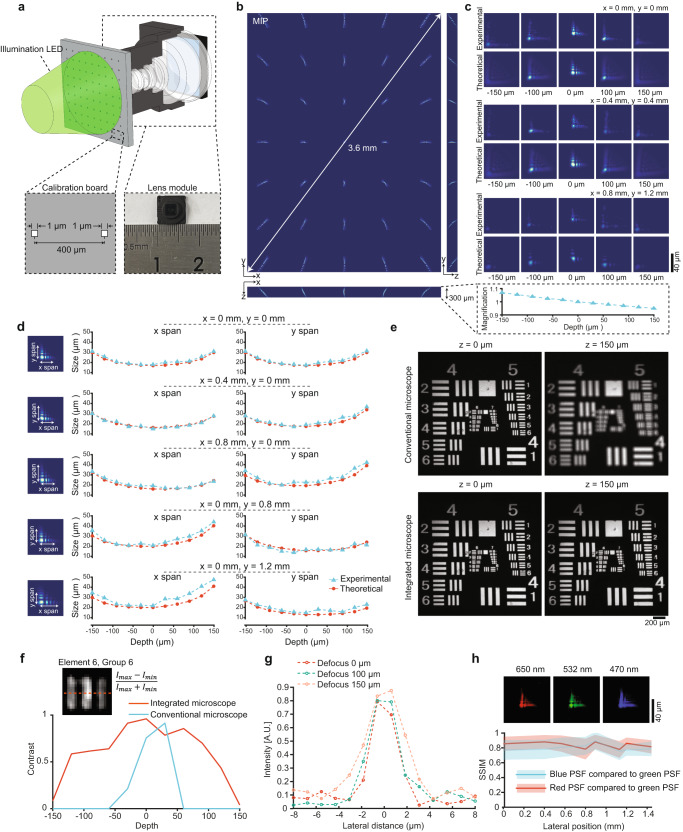
Evaluation of the integrated microscope with mass-producible fabrication. **a** The sketch of the PSF calibration setup of the integrated microscope with a customized 1-µm pinhole array through lithography. **b** Maximum intensity projections (MIPs) of captured PSF in *xy*, *xz*, and *yz* planes. The bottom plot shows the magnification changes across different depths. **c** Comparisons of simulated and experimental 3D PSFs at three different lateral positions. **d** Comparisons of the PSF size between simulations (red) and experiments (blue) at different lateral positions across a 300-µm axial range. **e** Characteristics of DOF extension through imaging the USAF-1951 resolution target. Top: imaging results with a conventional microscope (without DOE coding). Bottom: imaging results with a proposed integrated microscope with network restoration. **f** The contrasts of element 6, group 6 of the resolution target at different depths for the proposed integrated microscope (red) and conventional microscope (blue). **g** Full-width-half-maximums (FWHMs) of the 1-µm pinhole at different depths after network restoration (3.1 µm at *z* = 0 µm, 3.5 µm at *z* = 100 µm, and 4.8 µm at *z* = 150 µm). **h** The curves of structure similarity index (SSIM) calculated by PSFs of different illumination colors versus different lateral positions. Shaded areas: mean ± SD.

To verify the proposed framework in practice, we fabricated the integrated microscope through diamond turning and injection molding (“Methods”). Successive lens parts thus can be acquired at a low price (cheaper than $10 each) thanks to our plastic design, molding fabrication, and being free of cemented elements. To confirm the successful fabrications, we calibrated the proposed system through a customized 1-µm pinhole array written by lithography across a Φ3.6 mm FOV (Fig. 3a, Supplementary Fig. 9; “Methods”). As shown in Fig. 3b, the maximum intensity projection (MIP) of 3D distributed PSFs shows spread patterns in the margin of the FOV because of the finite conjugation of the system, where the experimental magnification highly correlates with the design value. Furthermore, shapes and intensity distributions of experimental PSF across different lateral and axial positions highly match those of the simulated PSFs, suggesting accurate modeling and fabrications (Fig. 3c).

The similarity between the designed PSFs and calibrated PSFs guarantees that the simulation-supervision network can effectively work on experimental data. To further verify it, we quantitatively compared the PSF size along the *x* and *y* directions in simulated and calibrated data. We found the simulated PSF size corresponded well with the experimental data at different depths and lateral positions across the entire sensor area (Fig. 3d). On the other hand, discrepancies during lens fabrication were inevitable. Considering the fabrication flaws of different instances are distributed in a centered manner, it is likely that the average similarity between the theoretical PSF and practical PSFs would be closest compared to the similarities between different instances of practical PSFs (Supplementary Fig. 14a). Additionally, the noise present during the calibration of practical PSFs can contribute to the convolution of the forward imaging process, which deviates from the physical process and introduces additional artifacts (Supplementary Fig. 14b). Hence, we employed theoretical PSFs for generating training pairs, as opposed to relying on experimentally calibrated PSFs. This approach ensures enhanced resilience against manufacturing imperfections during mass production and mitigates the impact of diverse noise sources encountered in the imaging process. Through numerical simulations, we proved that the trained neural network exhibited merely marginal performance degradation when confronted with the uppermost decenter (20 µm) and tip/tilt (0.1°) discrepancies potentially arising during the manufacturing (Supplementary Fig. 15), evincing the high robustness of the proposed neural network over the fabrication variability.

We conducted a qualitative assessment of the depth extension capabilities of the proposed integrated microscope using sample slides positioned at varying depths by a motorized stage (Supplementary Fig. 16). We found that the integrated microscope evidently improved resolution, contrast, and fidelity at defocused depths when compared to the ground truth in the focal plane (at *z* = 0 µm of the conventional microscope), without any apparent artifacts. We further confirmed the DOF extension by imaging a USAF-1951 resolution target placed at different axial planes (Fig. 3e, Supplementary Fig. 17). It is worth noting that the proposed neural network was trained on biological samples and microscopic samples from natural sources (such as insects, leaves, and flowers). Thus, the clearly restored USAF-1951 resolution target, which is substantially different from the training samples, additionally verified the generalization ability. We found both a conventional microscope and the integrated microscope achieved high-resolution chart images in the focal plane (*z* = 0 µm), but only the integrated microscope maintained that sharpness when the resolution target was largely defocused (*z* = 150 µm). A per-depth comparison further corroborated that the proposed integrated microscope achieved consistently high-quality images across various depths and various samples (Supplementary Fig. 17b).

We quantitatively compare the contrast of images obtained by both methods and find the proposed integrated microscope achieves overall higher contrast than the conventional microscope across the 300-µm depth range (Fig. 3f). With the simulation-supervision network, a 1-µm emitter can be restored tightly with full-width-half-maximum (FWHM) 3.1 µm at *z* = 0 µm, 3.5 µm at *z* = 100 µm, and 4.8 µm at *z* = 150 µm (Fig. 3g), suggesting the proposed microscope preserves high resolution across a large DOF.

To characterize chromatic aberrations of the system, we change the illumination spectrums of the calibration LED while fixing the pinhole array (“Methods”). We find that under blue, green, and red illuminations, the calibrated PSFs of three wavelengths are very similar with a structural similarity higher than 0.7 across the whole FOV (Fig. 3h), indicating that the chromatic aberrations are well corrected in the integration system. We proceeded to conduct a quantitative examination of the depth extension across various wavelengths by applying the trained neural network in each separate channel. We observed that the imaging performance of all three channels was closely aligned (Supplementary Fig. 18), signifying a consistent extended depth of field across multiple wavelengths.

### Integrated microscope in a cell phone enables information-rich imaging and portable diagnosis

**Fig. 4:**
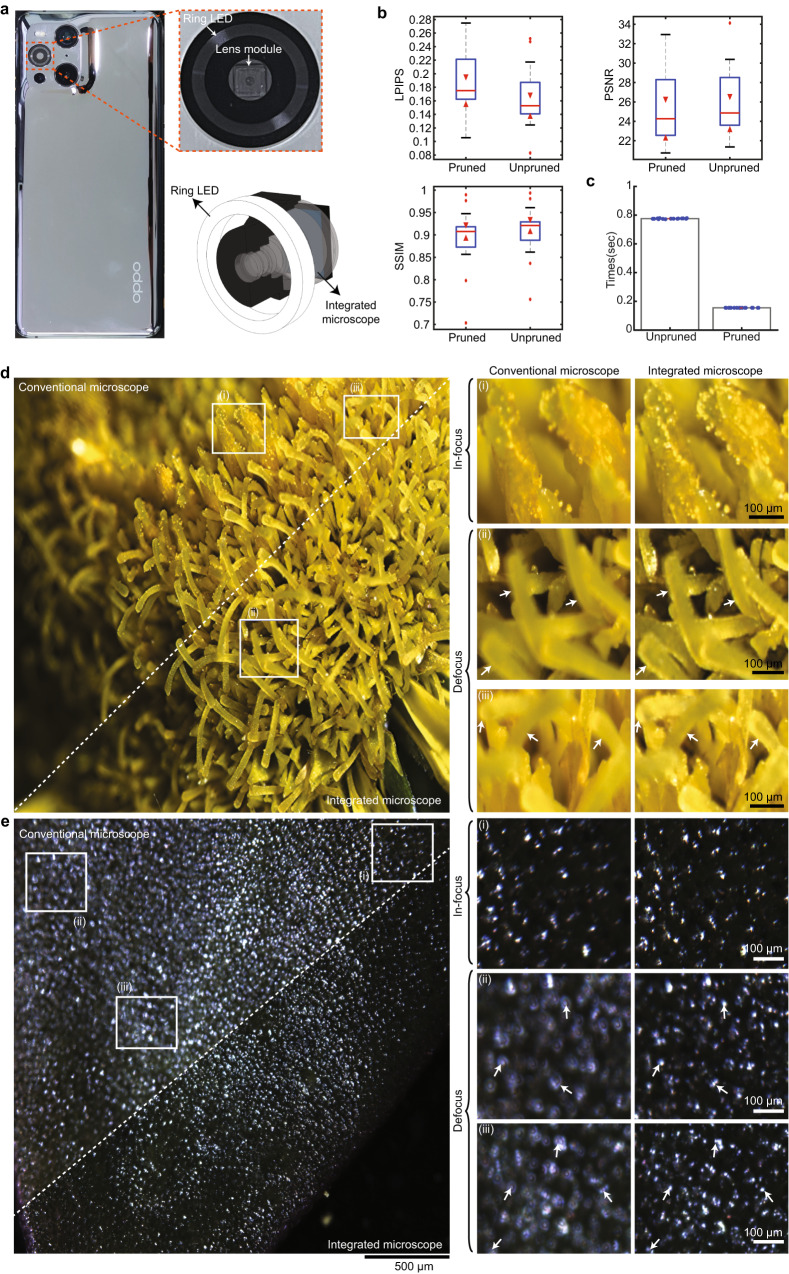
Integrated microscope equipped in a cell phone for real-time extended depth-of-field imaging. **a** Left, assemble the proposed integrated microscope in a cell phone. Right, the zoom-in panel shows the integrated microscope module (top) and 3D schematic diagram of the ring-shaped LED that is used for illumination (bottom). **b** After the introduction of network pruning, we reduced the network parameters by 78% with similar reconstruction performance regarding structure similarity index (SSIM), peak signal-to-noise ratio (PSNR), and perceptual loss (Learned Perceptual Image Patch Similarity, LPIPS). Central line inside the box: Median. Box: interquartile range. Whiskers: Maximum and minimum. Outliers: Individual data points. *n* = 19 samples. **c** Comparisons of the rendering time costs for *n* = 19 samples before and after network pruning. Data points are overlaid. Height of bars: Mean. Error bars: SD. **d** Left, comparisons of the captures obtained by the integrated microscope (bottom right) and a conventional microscope (top left) on yellow flowers. Right, zoom-in panels of the white dashed boxes on the left. White arrows indicate the structures which are hard to be resolved in a conventional microscope. Representative data from 53 samples. **e** The same as (**d**) but on samples of leaves. Representative data from 53 samples.

The proposed integrated microscope, with its small size and light weight, can be seamlessly integrated into a cell phone. As shown in Fig. 4a, the integrated microscope can be well equipped without largely bumped structures compared to previous mobile-phone-based microscopes^53,54^. The illumination is provided by a circular LED around the lenses. Besides the hardware integration, the optimized neural network used for reconstruction needs to be deployed in the cell phone for real-time visualization. To accommodate the processors in the mobile platform, we prune the network with 78% reduced parameters but nearly the same performance (Fig. 4b, Supplementary Fig. 10; “Methods”). The processing time of the pruned network is reduced by about 5 times (Fig. 4c).

To demonstrate the performance of the integrated microscope in the cell phone, we hold the cell phone and take pictures of yellow flowers (Fig. 4d). We find the proposed integrated microscope achieves clear features across multiple sites of the FOV in different depths. In comparison, a conventional microscope only achieves clear features within a small region that is near the focal plane, thereby relegating defocused elements to substantial blurriness. We further use the cell phone to image samples with different structures, including tilted *Rhizopus nigricans*, Paramecia, and plant root slices (Supplementary Fig. 11). We find that the proposed integrated microscope shows better performance than conventional microscopes across a much larger depth range for all the samples, suggesting achieving superior imaging quality and richer information with great generalizations. Besides, no color fringing is observed in all fields of captured samples, indicating full correction of chromatic aberrations.

**Fig. 5:**
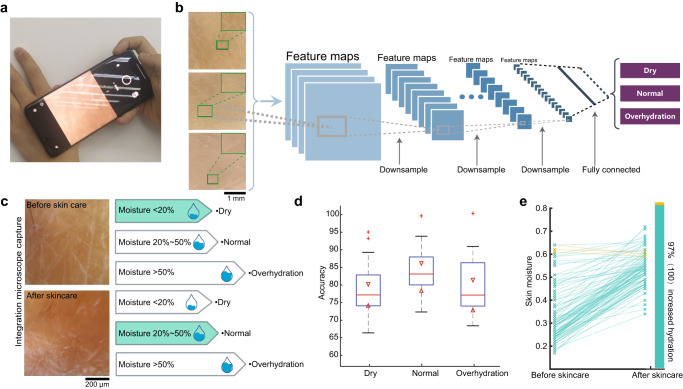
Portable health monitoring with the integrated microscope in a cell phone. **a** Illustration of using a cell phone with the integrated microscope built-in for skin hydration detection. **b** A convolution neural network is proposed to classify the output image of the integrated microscope into three classes, including dry, normal, and overhydration. **c** The proposed hydration detection algorithm resolves the features of dry skin which has many flakes (top). After skincare, the skin monitoring shows hydration back to normal (bottom). **d** The proposed hydration detection method achieves high accuracy compared to ground truths obtained by electrical hydration sensors for all three moisture levels. Central line inside the box: Median. Box: interquartile range. Whiskers: Maximum and minimum. Outliers: Individual data points. *n* = 28 samples. **e** The improvement of hydration identified by the integrated microscope after skincare (*n* = 100 tests).

The powerful imaging ability and miniaturized size of our integrated microscope shed new light on cell phone-based health monitoring. As an example, we show our integrated microscope helps monitor the hydration of the stratum corneum (Fig. 5a), a key factor in monitoring skin health. We develop a neural network with the input of deconvolved microscopic skin images and output of the moisture levels (dry, normal, and overhydration; Fig. 5b, c; “Methods”) to alarm decreased water content which impairs the natural desquamation process^55^. The neural network is further packaged into a customized application that quickly informs the user about the skin moisture level with high accuracy (Fig. 5d). With the reminder of the customized applications, users can timely detect skin dryness and intentionally dose lotions which effectively eases the dryness and maintains healthy skin conditions (Fig. 5e). An additional ablation study substantiated the necessity of high-resolution restoration of skin images by the proposed network to attain precise skin state detection (Supplementary Fig. 19). Compared to traditional ways that utilize an additional electronic device to monitor water content through conductance and capacitance, our integrated microscope in a cell phone readily provides quick suggestions on skin protections based on images. Much more complicated health monitoring and portable testing can be developed with the integrated microscope on cell phones in the future.

## Discussion

In summary, we propose an effective progressive optimization paradigm that fully leverages state-of-the-art optical design techniques and physics-based deep-learning reconstructions compositely. By first utilizing ray-tracing-based optimization to narrow down the searching space and then jointly optimizing optics and algorithms, we achieve an integrated microscope design that achieves the performance of tabletop microscopes but with five orders of magnitude smaller size and four orders of magnitude reduction in weight. The jointly optimized deep neural network is trained in a simulation-supervision manner that maximizes the domain similarity between simulation data and experimental capture and outperforms both traditional deconvolution algorithms and recently emerging unsupervised approaches. Together, the integrated microscope, after progressive optimizations, achieves 3 µm resolution across a FOV of Φ3.6 mm and a depth of field of 300 µm, which is about 10-fold larger than that of a typical microscope. The comprehensive optimization greatly reduces size and weight without compromising any performance, enabling integrations even in a cell phone for health monitoring. Further with the optimized network in cell phone processors, the integrated microscope can render the clear structures across the 300 µm depth range in real-time.

The optical design is heavily non-linear and is characterized by many local minima and steep ridges with many fabrication-related physical constraints (e.g., feasible central and edge thicknesses)^56^. Given these challenges, end-to-end lens optimizations in a top-down manner are only available in the image formation models that are either on simple wave optics models or on similar paraxial models^29^, providing simplistic solutions of a single lens surface in limited applications. Differently, our progressive optimization paradigm constrains the solution space into a feasible size and effectively avoids local optima through ray-tracing-based merits and refining an introduced diffractive optical element (DOE) and artificial intelligent algorithms to accomplish higher performance. In principle, the proposed optimization paradigm is scalable to any complex system (Supplementary Note 1), including the high-resolution miniaturized microscopic system in our case. Other optical systems like telescopes and surveillance systems can be extended through the proposed method with compressed size seamlessly. We acknowledge that our choice of a cubic phase distribution as the wavefront coding profile is not singular; indeed, alternate phase functions such as circular symmetric phase functions^57^ can also significantly augment the imaging depth of field. In accordance with scholarly articles, higher-order anti-symmetric phase masks and sinusoidal profiles can even yield high-quality images in the presence of substantial focus errors^44^. It is also worth noting that deep neural networks solely selected suitable candidates produced by traditional ray tracing, primarily due to the incompatibilities between traditional ray-tracing optimization and deep learning tools. The recently emerged differentiable ray-tracing technique^29^ offers the prospect of synergistically employing traditional high-efficiency ray optimization methods in conjunction with reconstruction algorithms, which is anticipated to evolve into a comprehensive design approach encompassing both traditional spherical and aspherical lenses as well as diffractive optical elements (DOEs).

Through the proposed optimization pipeline, we create the most compact mesoscope among ever-fabricated designs (Supplementary Tables 1 and 3; Supplementary Note 2). The introduction of ring-shaped LEDs takes advantage of the surrounding space of the trapezoid lens housing and is capable of fluorescent excitation via proper LEDs and coating without dichroic^9,27^. Our integrated microscope consists of plastic lenses without cemented elements for the capability of massive production. To achieve even more compact size and advanced performance, metasurfaces could be introduced to replace the plastic lenses with sub-micron thickness and over 80° of FOV angle^58^.

We have proven that a high-resolution integrated microscope that can be equipped in a cell phone stimulates new portable diagnoses in skin health without additional electronic devices. Other skin diseases like acne, pemphigus, and psoriasis can also be readily diagnosed through the proposed integrated microscope and corresponding intelligent algorithm in a single shot. Further with recently emerging virtual clinic services, patients can get convenient care only using a cell phone equipped with our integrated microscope at home. Moreover, the high-performance integrated microscope should facilitate probing microbiome^59^, blood-borne filarial parasites^60^, and waterborne pathogens^54^ on limited resources conditions and mitigating substantial threats to human health, and the portability opens up new possibilities for mobile assays for numerous conditions and diseases.

Capitalized on recent advances in genetically encoded calcium indicators (GECIs)^61^, our proposed integration microscope can be further extended for neuronal Ca^2+^ imaging in freely moving mice both at the cerebral cortex as well as the cerebellum and other brain regions^9,20,27,28^ (Supplementary Fig. 20). The ultra-compact form factor and minimized weight of our integrated microscope incur the least disturbance to animal motions compared to other head-mounted microscopes, thereby facilitating visualizing population-level microcirculation across different locomotor behaviors more naturally. Further combined with wire-free technologies^36^, the even more flexible integrated microscope will promote neuroscience research across widely used freely behaving assays, including fear conditioning and social interactions. The heavily optimized integrated microscope offers less costly, versatile, and stable solutions than the current optical apparatus in brain-imaging research.

In addition to potential use in behaving animals, the integrated microscope is a multipurpose instrument for various applications, including flow cytometry^9^, air quality monitoring^62^, and cancer screening^63^. For in vitro applications, the integrated microscope has the potential to achieve even higher throughput on a large scale through massively parallel strategies^12^ and is capable of being equipped with other instrumentation, such as incubators, thanks to the miniaturized sizes^9^. With upcoming GPU advances in improved speed, efficiency, and reduced size, integrated microscopes in intelligent platforms seem likely to facilitate the emerging paradigm of mobile analysis, screening, and diagnostic evaluations.

Lastly, we believe the proposed progressive optimization paradigm sheds new light on optical designs by harnessing the advantages of aspherical optics, computational imaging, and deep learning reconstructions in a complete pipeline. Catalyzed by the optimization formulas, the proposed integrated microscope sets a new record for miniaturized microscopes, which can facilitate diverse applications spanning from image-based mobile diagnostics to neural recording in freely behaving animals and beyond.

## Methods

### Preliminary design of the integrated microscope based on ray-tracing

We start designing the high-performance integrated microscope with ray-tracing merits which remarkably reduces the parameter searching space compared to brute-force deep-optics optimization. The system numerical aperture is set to be 0.16 for subcellular spatial resolution and fluorescence-capable energy collection efficiency, while the focal length remains 1 mm to ensure a compact system. It is obviously challenging to design a system with such a large aperture in a conjugate distance of only 6 mm by the traditional method. The parameters of the lenses do not exist independently but restrict mutually and are mainly constrained by the aberration correction efforts. Since the primary aberrations, such as spherical aberration, coma, astigmatism, and field curvature, are closely related to the aperture of the lens, optimization on such a relatively high-NA microscope faces substantial challenges.

First, we arranged the lens structure based on the principle of the Chevalier Landscape lens, which is the first widely used camera lens introduced after the invention of film-based photography, to correct aberrations. With reference to the structure model of the Chevalier Landscape lens, we set the aperture in the front of the imaging lens and made the aperture diameter smaller than that of the subsequent lens. In the optical path of the lens, the rays in normal and oblique incidence are separated by the frontmost aperture and then focused by different parts of the subsequent lenses so that the curvature of each lens can be adjusted to reduce aberrations, especially coma. On the other hand, the full correction of the aberrations from different incident fields requires the lens surface to be non-spherical.

Note that although a singlet spherical lens cannot achieve diffraction-limited focusing for different angles of incidence, adding more lenses in principle could provide more degrees of freedom to correct spherical aberration, coma aberration, astigmatism, and Petzval field curvature. However, this approach, combined with conventional lens manufacturing techniques, results in bulky imaging systems (Supplementary Fig. 1). This becomes tricky as the demand for portable and compact devices increases. Thereby, in our designs, multi-lens elements with non-spherical shapes are utilized for high optical performance. The surface profile for used aspherical lens can be expressed as

1 $$z=\frac{c{r}^{2}}{1+\sqrt{1-\left(1+k\right){c}^{2}{r}^{2}}}+{\alpha }_{1}{r}^{2}+{\alpha }_{2}{r}^{4}+{\alpha }_{3}{r}^{6}+{\alpha }_{4}{r}^{8}+{\alpha }_{5}{r}^{10}+{\alpha }_{6}{r}^{12}+{\alpha }_{7}{r}^{14}+{\alpha }_{8}{r}^{16}$$

where $c$ denotes the curvature, $r$ is the radial coordinate in lens units, $k$ is the conic constant, and ${\alpha }_{1}-{\alpha }_{8}$ are the coefficients. In addition, imperfect irradiances such as glare reflection, nonuniform distribution, and brightness level are considered as well. After the steps of initial building the surfaces, we iteratively optimized the proposed system in ZEMAX (OpticStudio) with the merit of resolution across all fields (up to 1.814 mm from the center) and wavelengths (400–700 nm).

### Extended depth-of-field design with a diffractive optical element

For the practical scenario in microscopy, the acquisition volume and the DOF are always required to be large enough to preserve the high reliability and robustness of the system. In standard microscopy, DOF is fundamentally coupled to lateral resolution $\triangle r$: ${DOF}\propto \frac{\lambda }{{{NA}}^{2}}\propto \frac{\Delta {r}^{2}}{\lambda }$. There is a fixed trade-off between depth-of-field (DOF) and lateral resolution—the higher the desired lateral resolution, the narrower the DOF. The conventional approach to increase the DOF is to decrease the NA, which corresponds to using a smaller aperture or a longer focal length. However, both scenarios have a side effect. A smaller aperture would lead to a poor optical throughput and thus a low signal-to-noise ratio outcome. On the other hand, a longer focal length increases the form factor of the devices, which contradicts the goal of miniaturization. Imaging optics with sufficient DOF while preserving satisfactory resolution is highly desirable.

In this regard, we propose a computational technique that breaks the above constraint and achieves a 10 times larger DOF while retaining cellular resolution, obviating the need for axial scanning and substantially reducing the imaging time required. The key factor is to optimize a diffractive optical element (DOE) placed near the microscope aperture and the subsequent deep learning-based reconstruction algorithm. In our case, the DOE is substantiated as a cubic phase plate (CPP) with the surface profile as ${{{{{\rm{\alpha }}}}}}\left({x}_{{DOE}}^{3}+{y}_{{DOE}}^{3}\right)$, where ${x}_{{DOE}}$ and ${y}_{{DOE}}$ constitute a right-angled coordinate system situated within the DOE plane, with the positive ${x}_{{DOE}}$ direction oriented toward the sagittal direction and the positive ${y}_{{DOE}}$ direction oriented toward the tangential direction. The single variable $\alpha$ is used to control the spread of the PSFs across different defocus and thereby controls the DOF.

We next optimized the best pupil modulation strength $\alpha$ through numerical evaluation. This needs to ensure that a trade-off is made between the following two points—the imaging characteristics within the designed range of defocus are as similar as possible, and some constraints are adopted to prevent an excessive optimization result and avoid difficulties in deblurring the captured images. Different from the conventional approach of obtaining the modulation strength based on diffraction-limited MTF in simulation, we set the MTF similarity across different axial depths of the CPP-equipped system as the merit function. Fisher information (FI) is an effective method that can be applied to evaluate the similarity of MTF, which can measure the variation of MTF encountering defocusing. If FI is zero, all defocused MTFs within the designed range of defocus are the same, indicating that the MTF is insensitive to defocus. Thus, the optimized aim of the phase parameters is to find the minimum FI. It should be noted that the MTF of the coded system cannot be too close to zero, which will cause a permanent loss of information and cannot be restored through post-processing. The situation that needs to be avoided more is that when the strong modulation is imposed, negative values (contrast reversal) occur. In order to keep the system within a safe range, it is necessary to prevent the $\alpha$ value from being over-optimized. The MTF threshold at the Nyquist frequency is greater than 0.1 to ensure that most information is above the noise floor and thus well recoverable.

To select the best $\alpha$ value, we individually conducted the aforementioned optimization for systems with $\alpha$ evenly distributed across 0.005 to 0.075, resulting in 15 optimized candidates. For each candidate, we separately trained a neural network (see “Network architecture and training details” section) and selected the preferential candidate based on the best reconstruction scores. We found that the optimal value of $\alpha$ was 0.03, which enabled a tenfold DOF increment nearly. In conventional microscopy with a standard objective, achieving subcellular lateral resolution (2–3 μm) restricts the DOF to ∼30 μm, which is almost one order of magnitude smaller than our optimized results. The extended DOF of the proposed system provides privileges in accommodating the variations in surface topography of freshly resected tissue surfaces. An additional example is delineated in Supplementary Note 1 and Supplementary Figs. 21–23.

### Chromatic aberration correction without cemented elements

Chromatic aberration is caused by the dispersion characteristics of the material or optical structure. Compared to an ideal lens that focuses a point in the object space on a point in the image space, light of different wavelengths generates focal spots at different spatial positions in a practical imaging system. This phenomenon deteriorates the performance of imaging systems under broadband illumination. In a microscope, dyes and labels that range a wide spectrum make chromatic correction necessary. In principle, chromatic aberration can be approximately corrected by using materials with complementary dispersion properties, as in an achromatic doublet. As one of the most commonly used optical elements in optical designs and engineering, an achromat cements a positive crown glass element (low refractive index) and a negative flint glass element (high refractive index) together. The compound lens brings at least two wavelengths of light to a common focus. However, this technique is cumbersome since the number of materials equals the number of wavelengths where the chromatic aberrations are minimized.

Instead, we presented an implementation of a non-cemented aspherical lens group made of two plastic optical materials (EP-9000 and ZEONEX_K22R&K26R_2017) for chromatic correction. Axial MTF and chromatic focal shift data in such a design are comparable with a system with cemented achromatic doublets. The secondary color was corrected mainly through the optimized aspherical surfaces since the two plastic materials alone are not sufficient to fully satisfy the dispersion diversity.

### Fabrications

After the optical design is finished, aspherical lenses are plastic molded, and the phase masks are fabricated through nanoimprint. All components (including plastic housing) are fabricated by Sunny optical technology. The manufacturing process typically involves a combination of CNC machining, injection molding, and surface coating. The lens barrel was machined from a solid block of aluminum using a CNC machine. The lens elements were injection molded using specialized equipment that was designed to produce high-quality optical polymers. Once the lens elements are produced, they are assembled into the lens barrel, and the entire assembly is coated with an anti-reflective coating to improve image quality. Nanoimprinting was opted as our chosen fabrication process in order to facilitate mass production. The mold for nanoimprinting was created using two-photon polymerization with the desired nanostructure patterns. The mold is then pressed onto the substrate surface, transferring a pattern from the mold to the substrate. Following this, the imprint was cured through UV light, and the mold and residual material were subsequently removed from the substrate.

Compared to a tabletop microscope (IX73, Olympus) with dimensions of 323 mm (W) x 475 mm (D) x 656 mm (H), which results in a total volume of 100,646,800 mm^3^, our integrated microscope features a size of 150 mm^3^, leading to a size reduction of 6.7 × 10^5^ and an overall volume reduction of 5 orders of magnitude.

### Network architecture and training details

Our network architecture employed pix2pix^64^ network, a GAN-based model that provides rich texture details for image restoration tasks. For the generator, our model was mainly based on the U-Net^65^ model, which was reported to have superior performance in microscopy tasks. In general, our generator network was composed of a U-Net encoder and decoder module. In the U-Net encoder module, four encoder blocks were used, where each block consisted of a 4×4 convolutional layer (stride=2) followed by a leaky rectified linear unit (LeakyReLU). In the U-Net decoder module, four symmetrical decoder blocks were used, where each block consisted of bilinear interpolation, 3×3 convolutional layer, followed by a ReLU. Considering the difficulty of restoring images with inconsistent PSF in the horizontal and axial direction, we used nine residual blocks after the encoder module to further strengthen the feature transformation ability of the network. Each residual block consisted of two 3×3 convolutional layers, followed by a ReLU and a shortcut connections component. As the core part of U-Net, a skip-connection architecture was used between the encoder module and the decoder module to fuse shallow features with deep features. For the discriminator, we adopted the standard PatchGAN model with 70 receptive fields from pix2pix. This discriminator architecture could penalize structure at the scale of local patches to encourage high-frequency details.

We used the GAN loss term, L2-norm loss term, and perceptual loss term^49^ for the loss function. Compared to pix2pix, we used the VGG19 model to extract features to compute an additional perceptual loss in feature level, which made the output look more realistic and accomplished better performance visually.

AdamW optimizer^66^ was used to optimize network training, with a learning rate of 0.0002 and exponential decay rates of 0.9 for the first moment and 0.999 for the second moment. We used a learning rate warmup for 10 epochs and then linearly decayed the learning rate over the course of training. We used graphics processing units (GPU) to accelerate the training and testing process. It took about 10 h to train our model for 300 epochs with a batch size of 16 on our training set (about 110 microscopy and daily life images with the size of 2160×2560×3) with 4 GPUs (NVIDIA TeslaV100, 16GB memory). In the training phase, we randomly cropped an image into 20 small image patches with a size of 512×512×3 such that we had 2200 images for training eventually. In the testing phase, we tested 19 images with the size of 2160×2560×3 directly.

### Shift-variant forward propagation model and training data acquisition

To train a restoration neural network for evaluating optical designs in the optimization stage, it is necessary to numerically simulate the blurred captures through the DOE-combined aspherical system. However, relatively large FOV (Φ3.6 mm) and high numerical aperture (NA 0.16) cause nonuniform point spread functions (PSFs) across the field-of-view (FOV), which precluded using traditional forward propagation models^15^. To manage this, we proposed a shift-variant forward model considering the PSF change with an optimized computing burden. An optical system with shift-variant PSF satisfies the general form of the following superposition formulation,

2 $$i\left(x,\, y\right)=\mathop{\sum}\limits_{u,v,z}s\left(u,\, v\right)p\left(u,\, v,\, x-u,\, y-v,\, z\right)$$

where $(u,\, v)$ and $(x,\, y)$ represent object and image space coordinates, respectively, and $z$ represented different depths. Point $s(u,\, v)$ generates a corresponding PSF $p(u,\, v,\, x,\, y,\, z)$ which relates to field position $(u,{v})$ instead of offset position $(x-u,\, y-v)$.

Given the difficulties that querying all PSFs corresponding to all field points $(u,\, {v})$, it is necessary to reduce the dimension of PSF matrix $p(u,\, v,\, x,\, y,\, z)$. One effective step of characterizing the PSF change within large FOV in lower dimensions is matrix factorizaton^37,48^, that is, to model the PSF as the weighted sum of a set of bases ${h}_{i}(x,\, y)$ and corresponding coefficient maps ${w}_{i}\left(u,\, v\right)$ for encoding the spatial variability,

3 $$p\left(u,\, v,\, x,\, y,\, z\right)=\mathop{\sum }\limits_{i}^{N}{w}_{i}\left(u,\, v,\, z\right){h}_{i}\left(x,\, y,\, z\right)$$

where $N$ is the number of effective bases and ${h}_{i}(x,\, y)$ satisfies the shift-invariant property. In practice, we calibrated ${M}(N\le M=49)$ field points for each depth $z$ in total, resulting in a collection of PSF $\left\{p\left(u,\, v,\, {x}_{i},\, {y}_{i},\, z\right)\right\},1\le {i}\le M$. The numerical or experimental PSF $\left\{p\left(u,\, v,\, {x}_{i},\, {y}_{i},\, z\right)\right\}$ are downsampled, tailored, vectorized, and merged into a PSF matrix ${{{{{\bf{P}}}}}}\in {{\mathbb{R}}}^{{ab}\times M}$ for total ${ab}$ sensor pixels. Similarly, ${h}_{i}(i=1,\ldots,N,N\le M)$ was expressed as ${{{{{\bf{H}}}}}}{{{\in }}{\mathbb{R}}}^{{ab}\times N}$, and ${w}_{i}(i=1,\ldots,N,N\le M)$ was expressed as ${{{{{{\bf{W}}}}}}{\mathbb{\in }}{\mathbb{R}}}^{N\times M}$. Then the above factorization process can be translated into an optimization problem known as non-negative matrix factorization^67^. We described ${{{{{{\bf{P}}}}}}}^{{ab}\times M}$ as ${{{{{{\bf{H}}}}}}}^{{ab}\times N}{{{{{{\bf{\times }}}}}}{{{{{\bf{W}}}}}}}^{N\times M}+{{{{{{\bf{E}}}}}}}^{{ab}\times M}$ where ${{{{{\bf{E}}}}}}\in {{\mathbb{R}}}^{{ab}\times M}$ donates error matrix between truth and estimation. In other words, ${{{{{\bf{H}}}}}}$ and ${{{{{\bf{W}}}}}}$ can be simultaneously optimized by solving the equation

4 $$\hat{{{{{{\bf{H}}}}}}},\, \hat{{{{{{\bf{W}}}}}}}=\mathop{{{{{{\rm{arg}}}}}}\,\,{{\min }}}\limits_{{{{{{\bf{H}}}}}},{{{{{\bf{W}}}}}}}{{{{{{\rm{||}}}}}}{{{{{\bf{H}}}}}}\times {{{{{\bf{W}}}}}}-{{{{{\bf{P}}}}}}{{{{{\rm{||}}}}}}}_{2}^{2}$$

We used Hierarchical Alternating Least Squares (HALS) algorithm^67^ to solve the above problem. For the purpose of reducing color fringing caused by nonuniform coefficient maps across channels, we modified the algorithm such that the coefficient maps are uniform across channels for each depth.

After the above simplification, the complete shift-variant forward propagation model can be written as:

5 $$i\left(x,\, y,\, z\right)=\mathop{\sum }\limits_{i=1}^{N}\mathop{\sum}\limits_{u,\,v}s\left(u,\, v\right){w}_{i}\left(u,\, v\right){h}_{i}\left(x-u,\, y-v,\, z\right)$$

With the simplification by using the convolution operator, the above formula can be further written as:

6 $$i\left(x,\,y\right)=\mathop{\sum }\limits_{i=1}^{N}\left\{\left(s\left(u,\, v\right)\times {w}_{i}\left(u,\, v\right)\right)*{h}_{i}\left(u,\, v\right)\right\}\left[x,\, y\right]$$

where $*$ indicates discrete convolution operator and can be implemented by FFT.

We utilized a motorized stage (M-VP-25XA-XYZL, Newport) and a tabletop microscope with 5× objective (MPLFLN 5X, Olympus) to capture both microsection and tiny objects as samples. The sample was first focused in the focal plane and motorized scanned axially across −150 µm to +150 µm at a step of 10 µm to form focal stacks, which were further used to generate training pairs.

### Deconvolution reconstruction algorithm

To restore clear images from coded captures, deconvolution algorithms are widely used^68^. The deconvolution problem regularized by total-variation distance can be represented in the following way:

7 $${{{{{\bf{S}}}}}}{{{{{\boldsymbol{=}}}}}}\mathop{{{{{{\rm{arg}}}}}}\,\,{{\min }}}\limits_{\,}{{{{{{\rm{||}}}}}}A\times {{{{{\bf{S}}}}}}-{{{{{\bf{I}}}}}}{{{{{\rm{||}}}}}}}_{2}^{2}+{\lambda }_{{TV}}{{{{{{\mathcal{D}}}}}}{{{{{\bf{S}}}}}}{{{{{\rm{||}}}}}}}_{1}$$

where $A$ System transformation function, ${{{{{\bf{S}}}}}}$ is the sample, ${{{{{\bf{I}}}}}}$ is the input degraded capture, ${{{{{\mathcal{D}}}}}}$ donates total variation (TV) regularization by gradient, and ${{{{{{\rm{\lambda }}}}}}}_{{{{{{\rm{TV}}}}}}}$ is an adjustable regularization parameter. We applied the alternating direction method of multipliers (ADMM)^69^ to solve the above equation.

On the other hand, the above optimization requires the PSFs to be uniform across the fields, which contradicts the facts in our mesoscopic imaging system. We thereby utilized a modified Richardson-Lucy deconvolution algorithm with TV regularization^48^ regarding the aforementioned shift-variant forward propagation model. The $k$-th iteration is as follows:

8 $$T{V}_{k}=\frac{1}{1-{\lambda }_{{TV}}\,\cdot {div}\left[\frac{\nabla {S}_{k}}{\left|\nabla {S}_{k}\right|}\right]}$$

9 $${I}_{k}^{*}=\mathop{\sum }\limits_{i=1}^{N}{{{{{\mathcal{F}}}}}}\left\{{p}_{i}\right\}{{{{{\mathcal{\cdot }}}}}}{{{{{\mathcal{F}}}}}}\left\{{a}_{i}\cdot {S}_{k}\right\}$$

10 $${R}_{k}=\frac{I}{{{{{{{\mathcal{F}}}}}}}^{-1}\left\{{I}_{k}^{*}\right\}}$$

11 $${E}_{k}^{*}=\mathop{\sum }\limits_{i=1}^{N}{{{{{\mathcal{F}}}}}}\left\{{p}_{i}\right\}{{{{{\mathscr{\cdot }}}}}}{{{{{\mathcal{F}}}}}}\left\{{a}_{i}\cdot {R}_{k}\right\}$$

12 $${S}_{k+1}=T{V}_{k}\cdot {{{{{{\mathcal{F}}}}}}}^{-1}\left\{{E}_{k}^{*}\right\}\cdot {S}_{k}$$

where ${{{{{\mathcal{F}}}}}}$ and ${{{{{{\mathcal{F}}}}}}}^{-1}$ were Fourier and inverse Fourier transforms, respectively, * represented the variable in the complex domain, and the value of ${\lambda }_{{TV}}$ was set as 0.00015. All deconvolution algorithms are implemented using MATLAB.

### PSF calibration

We fabricated a 1-μm pinhole array in a 1-mm thick glass slide through binary lithography. The glass slide that contained the pinhole array was then mounted in a customized holder that matched the cell phone. The fabricated lenses were mounted before a GC5035 sensor that was already been embedded inside an OPPO Find X3 cell phone for calibration. To calculate the size of the PSF, we first cropped the PSF in one site and binarized the PSF by the threshold that equals 10% of the maximum intensity of the PSF. We then picked up the maximum component through *bwconncomp* in MATLAB and calculated the size in the *x* and *y* directions. We illuminated the sample with LEDs with different wavelengths (M470L5 for blue illumination, M530L4 for green illumination, and M590L4 for red illumination. All LEDs are from THORLABS) while keeping the pinhole array fixed at the same position. The colorful PSFs thus were acquired, visualized by ImageJ, and evaluated for chromatic aberration measurement.

### Network pruning and migration in mobile phone

Considering the limitation of computational cost, memory usage, and real-time requirement in mobile devices, we designed a lightweight version for our network by pruning the number of channels in our generator network. For the U-Net encoder module, the origin output channels of four encoder blocks are 128, 256, 512, and 512, respectively. Now it becomes 32, 64, 128, and 256. The decoder module also makes symmetrical changes to keep the U-shaped structure. The number of output channels in nine residual blocks module correspondingly becomes 256. When migrating our model to mobile devices, we used the sigmoid activation function at the end of the generator instead of tanh because of the acceleration of mobile phone processors. All training procedures were accomplished on desktop PCs. The restoration of the captured image (i.e., inference through the trained network) was carried out through the APP using the computational sources available on the mobile phone. After capturing an image using the proposed integrated microscope, a low-resolution reconstruction will be produced through deconvolution for preview purposes. In the background, the network will restore the high-resolution image, which will then be stored in the phone gallery. The typical computation time on the APP side for the pruned network was 1729.3 ms.

### Skin moisture measurement

One 35-year-old male volunteered to be tested with informed consent. Initially, we employed our proposed integrated microscope to capture images across multiple regions of the volunteer’s hands and lower arms, subsequently followed by the measurement of skin moisture levels at identical locations using a portable skin tester (Pocreation). The paired data from the integrated microscope capture and the skin moisture values were utilized to construct a customized skin moisture detection application (details outlined in subsequent sections). Measurements were reiterated post-application of skincare (Vitamin C cream, Elastalift; a single drop per location). Our research complies with all relevant ethical regulations overseen by the Committee on Ethics of Tsinghua University.

### Customized skin moisture detection applications

We employed the MobileNet-V2 to complete the skin moisture detection task in a cell phone. It is a lightweight convolutional neural network for classification and segmentation tasks. The model used depth-wise separable convolution to reduce computation and the number of parameters, making it possible to deploy this model directly on mobile devices. Compared to Mobilenet-V1, it used inverted residuals and linear bottlenecks to get better performance.

For the loss function, we used the Cross-Entropy (CE) loss. We resized the input image size to 512 and trained the model for 240 epochs with a batch size of 128 on our training set (about 9000 skin images). Adam optimizer was used to optimize network training, with a learning rate of 0.0002 and exponential decay rates of 0.9 for the first moment and 0.999 for the second moment. The network was trained on a server and then migrated into the cell phone for portable diagnosis.

### Image quality metrics

#### Structure similarity (SSIM)

Structure similarity index (SSIM) is a widely used full-reference metric for the assessment of the visual quality of images and remote sensing data. We called *ssim* function in MATLAB to calculate the similarity between PSFs from different spectrums or the similarity between reconstruction images and ground-truth images.

#### Peak signal-to-noise-ratio (PSNR)

Peak signal-to-noise ratio (PSNR) is an engineering term for the ratio between the maximum possible power of a signal and the power of corrupting noise that affects the fidelity of its representation. We called *psnr* function in MATLAB to calculate the similarity between reconstruction images and ground-truth images.

#### Perceptual loss

Learned Perceptual Image Patch Similarity (LPIPS)^70^. It is a perceptual similarity metric that is based on deep features extracted from a neural network. It can compute a “perceptual distance,” which measures how similar two images are in a way that coincides with human judgment. Compared to PSNR and SSIM, the result of the LPIPS metric is more in line with human perception. In our work, we used the pretrained AlexNet^71^ to extract image features and compute the “perceptual distance” between output images and label images. The lower the value of this evaluation metric, the higher the perceptual similarity.

### Reporting summary

Further information on research design is available in the Nature Portfolio Reporting Summary linked to this article.

## Supplementary information

Supplementary Information

Description of additional supplementary files

Supplementary Software 1

Reporting Summary

## Data Availability

Demo datasets, expected outputs from demos, as well as pretrained model weights, are available for download at https://zenodo.org/record/7950911 (ref. ^41^, 10.5281/zenodo.7950911). Further data that support the findings of this study are available from the corresponding author upon request.

## Code availability

The custom code that contains the deconvolution neural network and simulation dataset generation is available in Supplementary Software 1 under an open-source license permitting not-for-profit research use (see file LICENSE.txt). The code is also available for download at https://zenodo.org/record/7953526. Future updates to the code will be published at https://github.com/yuanlong-o/mobilephone_EDOF.
