# Supplementary material for: Large depth-of-field ultra-compact microscope by progressive optimization and deep learning
Source: Nat Commun. 2023 Jul 11;14:4118. doi: 10.1038/s41467-023-39860-0 (PMC10336131; doi:10.1038/s41467-023-39860-0)
Supplement: Supplementary file 1 — Supplementary Information [file 41467_2023_39860_MOESM1_ESM.pdf]

Large depth-of-field ultra-compact microscope by progressive optimization and deep learning

Yuanlong Zhang[1,2,3,4*], Xiaofei Song[5*], Jiachen Xie[1,2,3,4*], Jing Hu[6*], Jiawei Chen[7], Xiang Li[7], Haiyu Zhang[7], Qiqun Zhou[7], Lekang Yuan[8], Chui Kong[9], Yibing Shen[6], Jiamin Wu[1,2,3,4‡], Lu Fang[10‡], and Qionghai Dai[1,2,3,4‡]

[1] Department of Automation, Tsinghua University, Beijing 100084, China
[2] Institute for Brain and Cognitive Sciences, Tsinghua University, Beijing 100084, China
[3] Beijing Key Laboratory of Multi-dimension & Multi-scale Computational Photography (MMCP), Tsinghua University, Beijing 100084, China
[4] Beijing Laboratory of Brain and Cognitive Intelligence, Beijing Municipal Education Commission, Beijing 100084, China
[5] Tsinghua Shenzhen International Graduate School, Tsinghua University, Shenzhen 518055, China
[6] State Key Laboratory of Modern Optical Instrumentation, Zhejiang University, Hangzhou 310027, China
[7] OPPO Research Institute, Shenzhen 518101, China
[8] Tsinghua-Berkeley Shenzhen Institute, Tsinghua University, Shenzhen 518055, China
[9] School of Information Science and Technology, Fudan University, Shanghai 200433, China
[10] Department of Electronic Engineering, Tsinghua University, Beijing 100084, China

[*] These authors contributed equally to this work
[‡] Correspondence should be addressed to J.W. (wujiamin@tsinghua.edu.cn), L.F. (fanglu@tsinghua.edu.cn), and Q.D. (qhdai@tsinghua.edu.cn)

Supplementary Information Table

**Supplementary Figure 1**	Aspherical lens system effectively reduces the system length with high performance.
**Supplementary Figure 2**	Evaluation diagrams of the optical design of the proposed integrated microscope.
**Supplementary Figure 3**	Forward model for the generation of synthetic dataset.
**Supplementary Figure 4**	Comparisons between the synthetic coded images and the experimental coded images.
**Supplementary Figure 5**	Simulation-supervision network effectively recovers coded images.
**Supplementary Figure 6**	Shift-variant deconvolution algorithm outperforms uniform deconvolution algorithm.
**Supplementary Figure 7**	Comparisons of refocusing method with simulation-supervision method in the integrated microscope.
**Supplementary Figure 8**	Comparisons of unsupervised method with simulation-supervision method in the integrated microscope
**Supplementary Figure 9**	Spatially nonuniform PSF calibrations across a large FOV of the integrated microscope.
**Supplementary Figure 10**	Pruned network has comparable fidelity to unpruned network with reduced computational costs.
**Supplementary Figure 11**	Comparisons of conventional microscope and the proposed EDOF integrated microscope equipped on a cell phone.
**Supplementary Figure 12**	Comparison of the final output of the integrated microscope scales with different coefficients (α) of the cubic phase plate (CPP).
**Supplementary Figure 13**	Illustrations of generating training pairs through the simulation supervision strategy.
**Supplementary Figure 14**	Comparison of imaging restoration results by the theoretical PSF-driven neural network and the calibrated PSF-driven neural network.
**Supplementary Figure 15**	Comparison of imaging restoration results by the theoretical PSF-driven neural network across various instances of the proposed integrated microscope.
**Supplementary Figure 16**	Comparison of imaging results of biological samples by the proposed microscope and a traditional microscope.
**Supplementary Figure 17**	Comparison of imaging results of a USAF1951 resolution chart through conventional microscope and integrated microscope in detail.
**Supplementary Figure 18**	Assessment of the impact of chromatic aberration on the extension of depth.
**Supplementary Figure 19**	Ablation study of skin state diagnosis related to the resolution of the skin images.
**Supplementary Figure 20**	Simulation of the integrated microscope in capturing cortical neuron activities.
**Supplementary Figure 21**	Optical characteristics of an optimized 10x microscopic systems without diffractive optical elements (DOE).
**Supplementary Figure 22**	Optical characteristics of an optimized 10x microscopic system with diffractive optical elements (DOE)

**Supplementary Figure 23**	Comparison of the final output of the integrated microscope scales with different coefficients (α) of the cubic phase plate (CPP) in a 10x microscopic system.
**Supplementary Table 1**	Reproducible workflow of the proposed design approach.
**Supplementary Table 2**	Comparison of the proposed integrated microscope with Foldscope and Cellscope.
**Supplementary Table 3**	Comparison of the required computational sources of deep optics optimization and the proposed optimization.

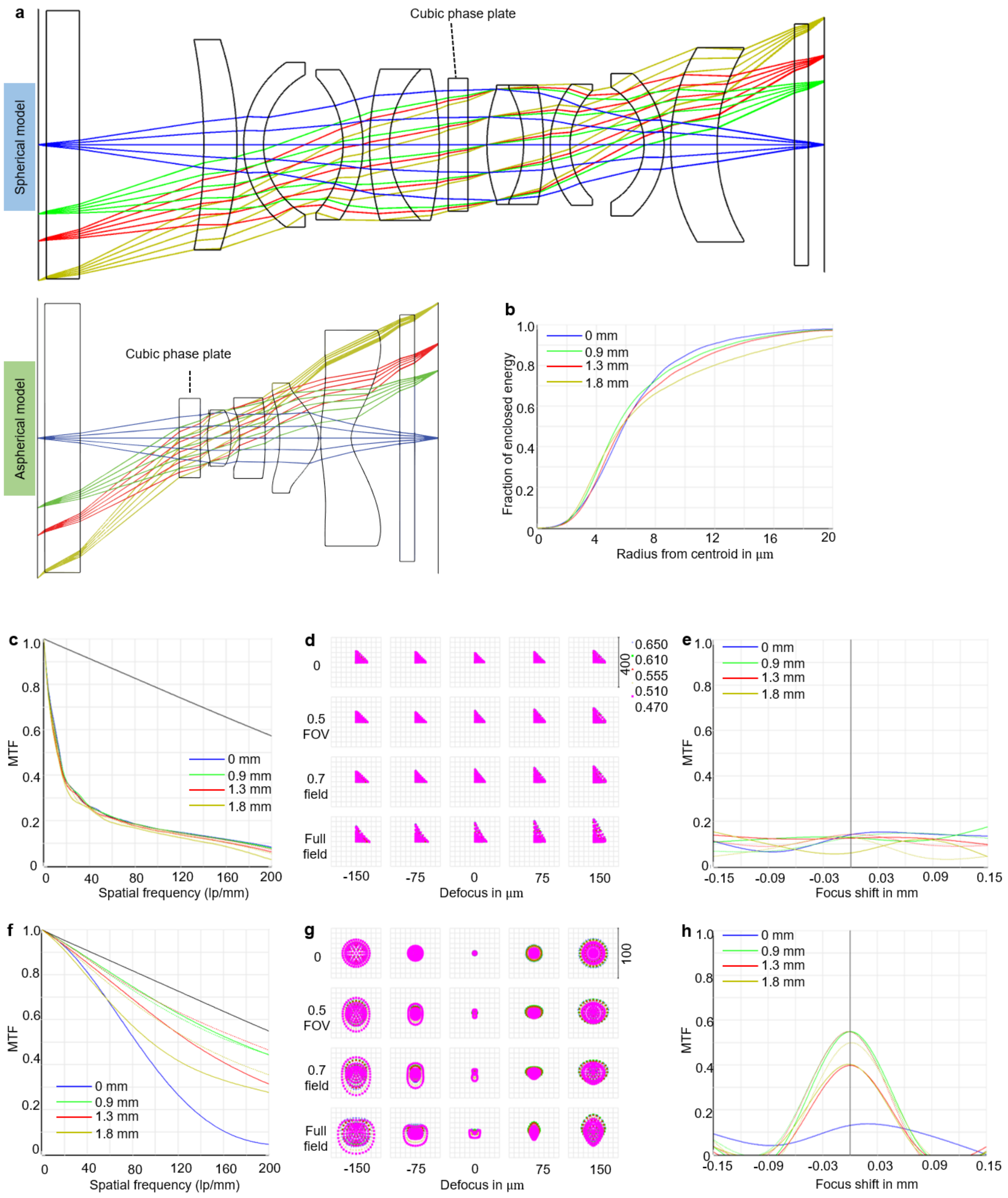

Supplementary Fig. 1. Aspherical lens system effectively reduces the system length with high performance.

a. The proposed optical design with aspherical plastic lenses (bottom) significantly reduces the size and weight of the system with spherical lenses (top) when the performance of both is similar. The conjugate distance (11.735 mm) of the spherical lens system is twice that of the aspherical lens system.

b. Encircled energy analysis of the optimized spherical system at different lateral positions of the field of view (FOV).

c-**e**. Evaluation diagrams of the spherical system with a cubic phase plate (CPP) inserted. The evaluations include modulation transfer function (MTF) at different lateral positions (**c**), spot diagram at different focal planes and different lateral positions (**d**), and the MTF along different focal planes at different lateral positions (**e**).

f-**h**. The diagrams corresponding to (**c**-**e**) without the CPP inserted in the optimized spherical system.

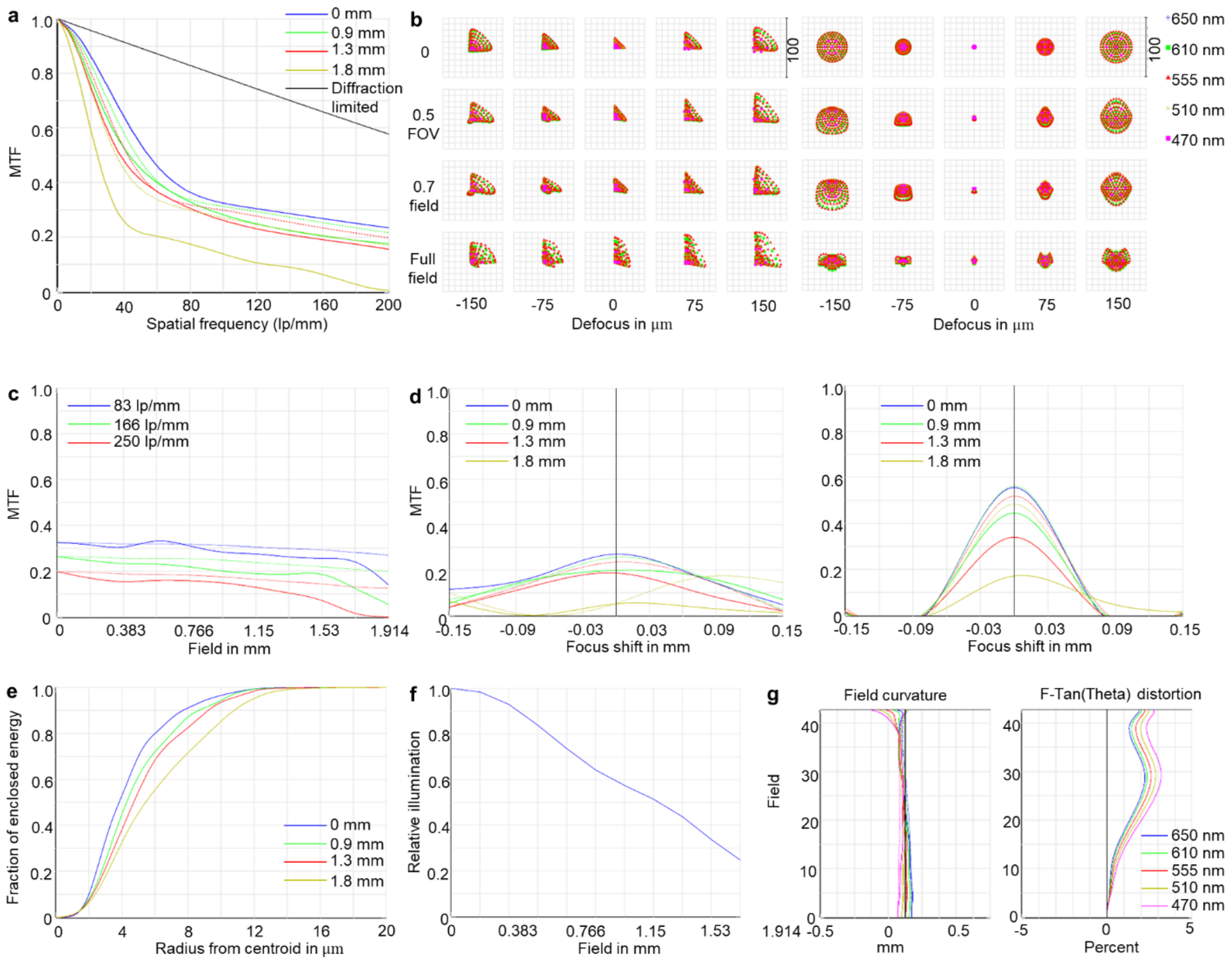

Supplementary Fig. 2. Evaluation diagrams of the optical design of the proposed integrated microscope.

a. The modulation transfer function (MTF) curves across different spatial frequencies for different lateral field positions. The black solid line indicates the diffraction limit.

b. Spot diagrams at different focal planes and lateral positions for the same design with cubic phase plate (CPP, left)and without CPP (right). Different color corresponds to different wavelengths.

c. MTF across different field positions at spatial frequencies of 83, 166 and 250 lp/mm (corresponding to spatial resolution of 6 µm (blue lines), 3 µm (green lines) and 2 µm (red lines).

d. MTF at different focal planes with a spatial frequency of 166 lp/mm for the same design with CPP (left) and without CPP (right).

e. Diffraction Encircled energy across different field radius.

f. Relative illumination intensity across different field positions.

g. Field curvature and distortion across different field positions of different wavelengths.

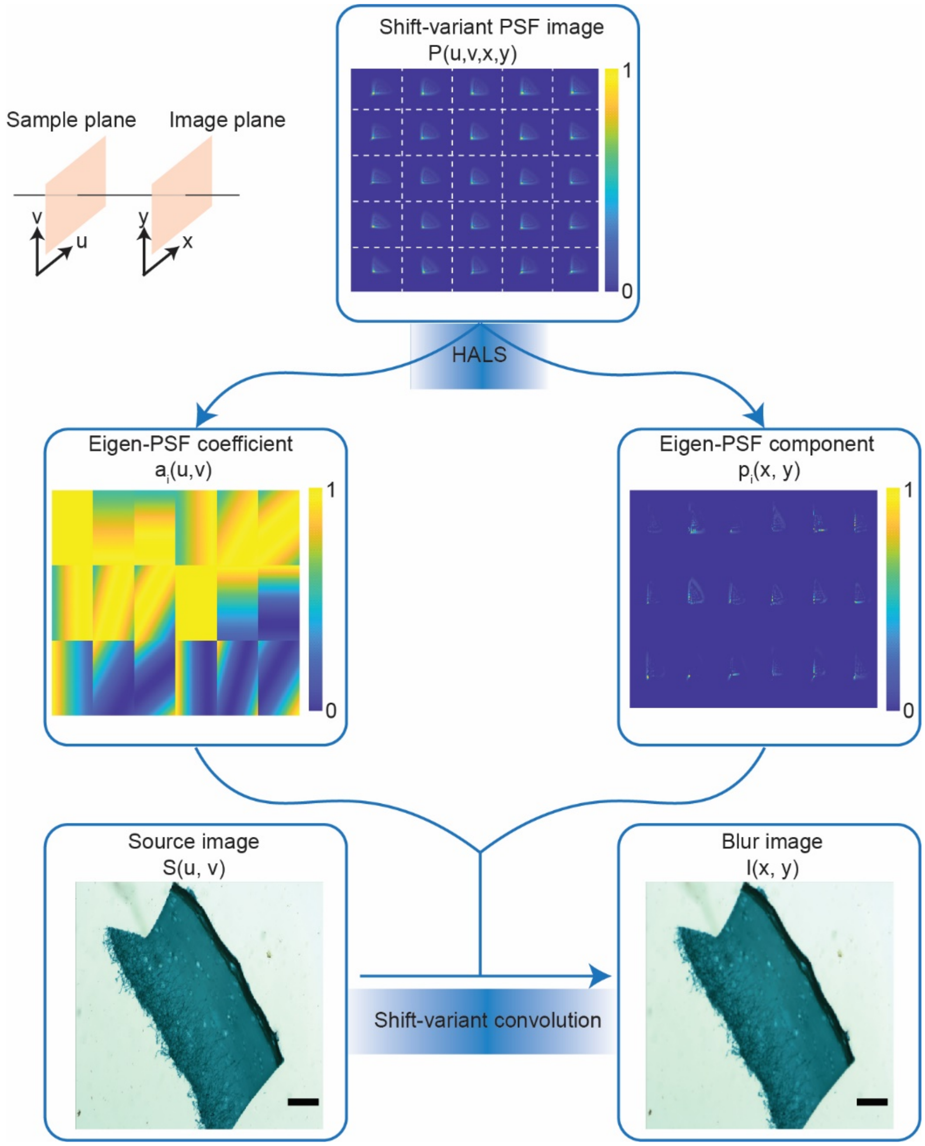

Supplementary Fig. 3. Forward model for the generation of synthetic dataset.

We consider the point spread function (PSF) of our optical system as the sum of a set of orthogonal bases $p_i(x, y)$ multiplexed with the corresponding coefficient map $a_i(u, v)$ for encoding the spatial variability. Given the spatial variant PSF $P(u, v, x, y)$ simulated from large fields, we generate the eigen-PSF component $p_i(x, y)$ and corresponding coefficient $a_i(u, v)$ utilizing modified Hierarchical Alternating Least Squares (HALS) algorithm. We share the same coefficient map $a_i(u, v)$ for different color channels to suppress chromatic aberration during image reconstruction. Finally, we use shift-variant convolution formula to produce source and blur image pairs (Methods). Scale bar: 500 μm.

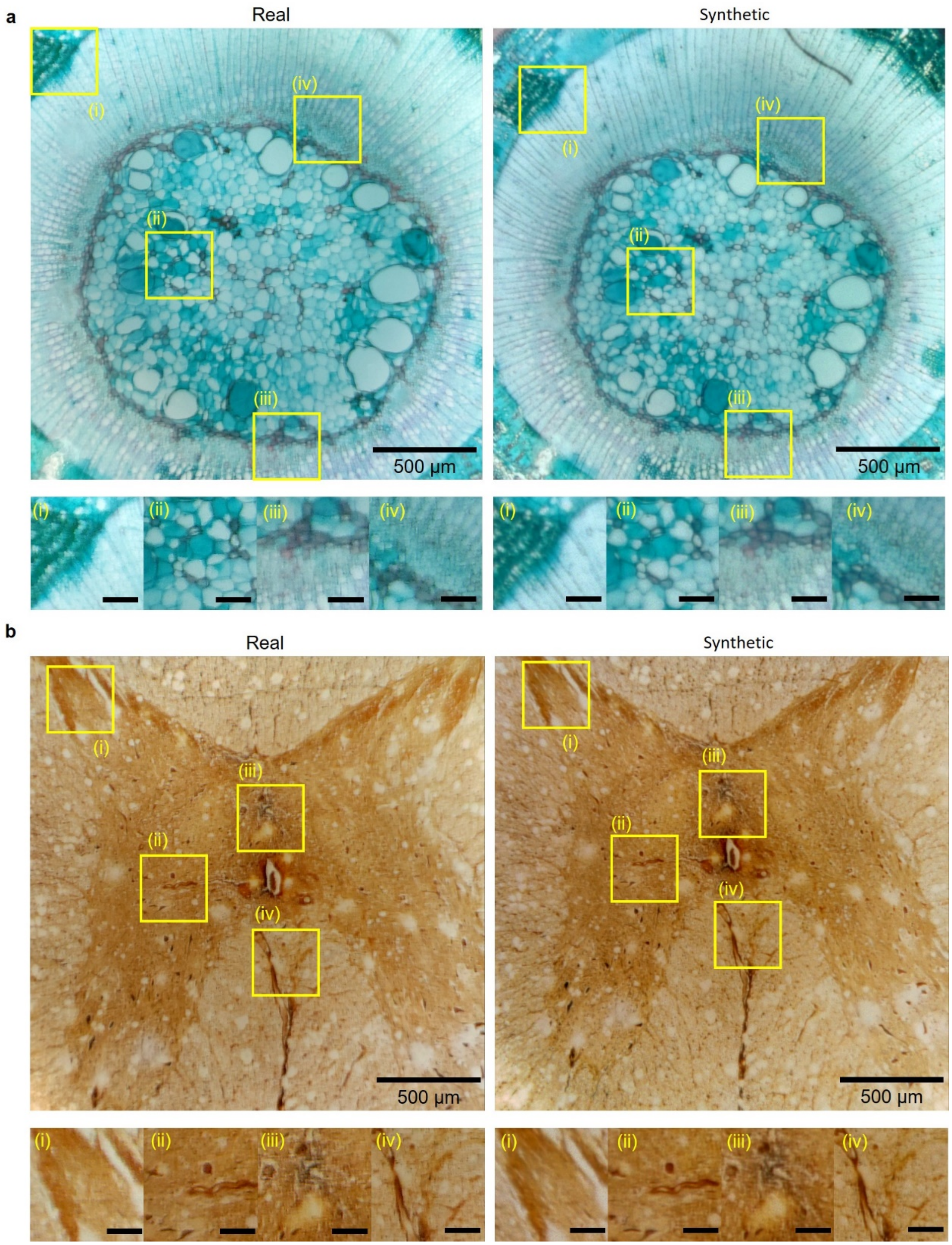

Supplementary Fig. 4. Comparisons between the synthetic coded images and the experimental coded images. **a, b**. Two different samples (woody dicot stem cross section and spinal cord transection, respectively) are shown to demonstrate the similarity between the experimental data (left, scale bar: 500 µm) and the synthetic data (right, scale bar: 500 µm). Zoom-in areas from (i) to (iv) are listed in the bottom for more detailed comparisons (scale bar: 100 µm). Representative data from 106 samples.

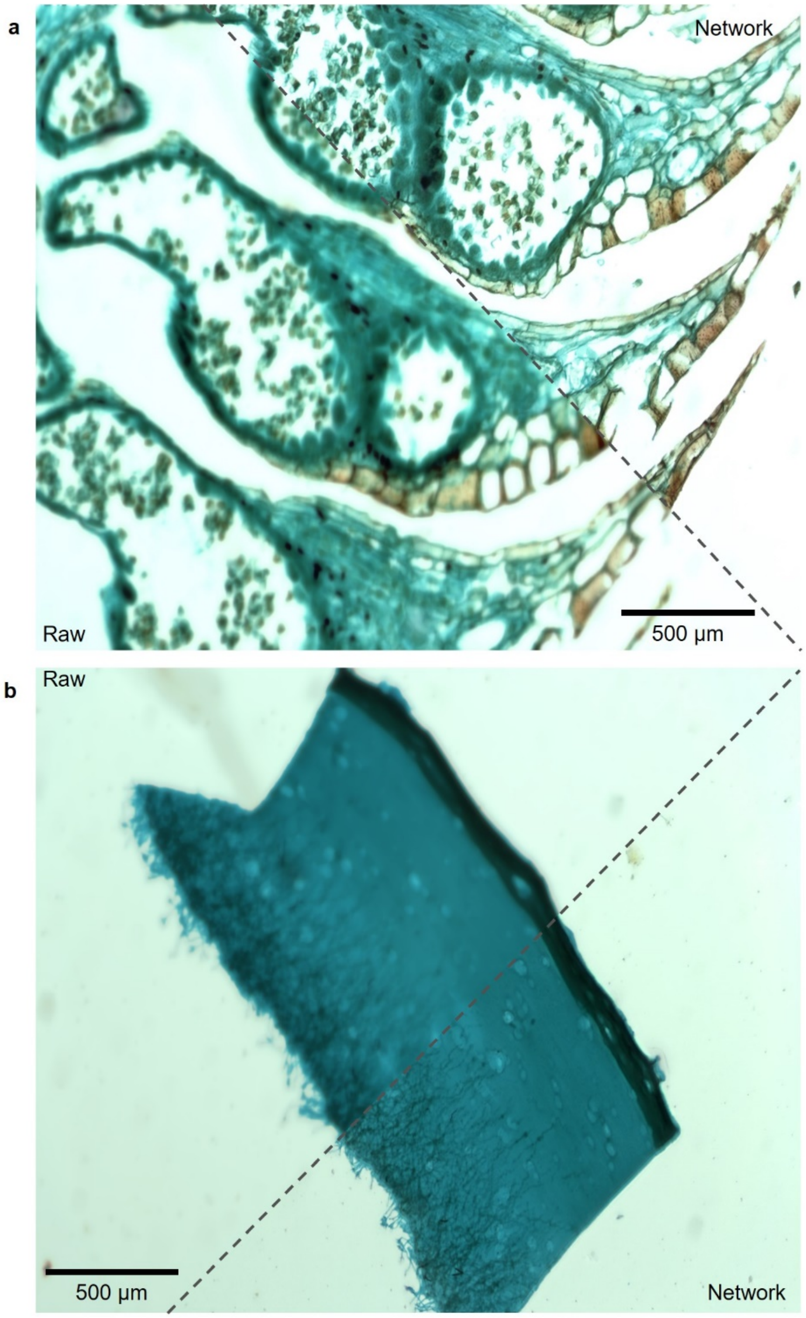

Supplementary Fig. 5. Simulation-supervision network effectively recovers coded images.

a, b. Two different sliced samples (top: pine male globose fruit, bottom: Penicillium, respectively) are displayed to indicate simulation-supervision network obviously gains clearer reconstructions from the raw images (scale bar: 500 µm). Representative data from 122 samples.

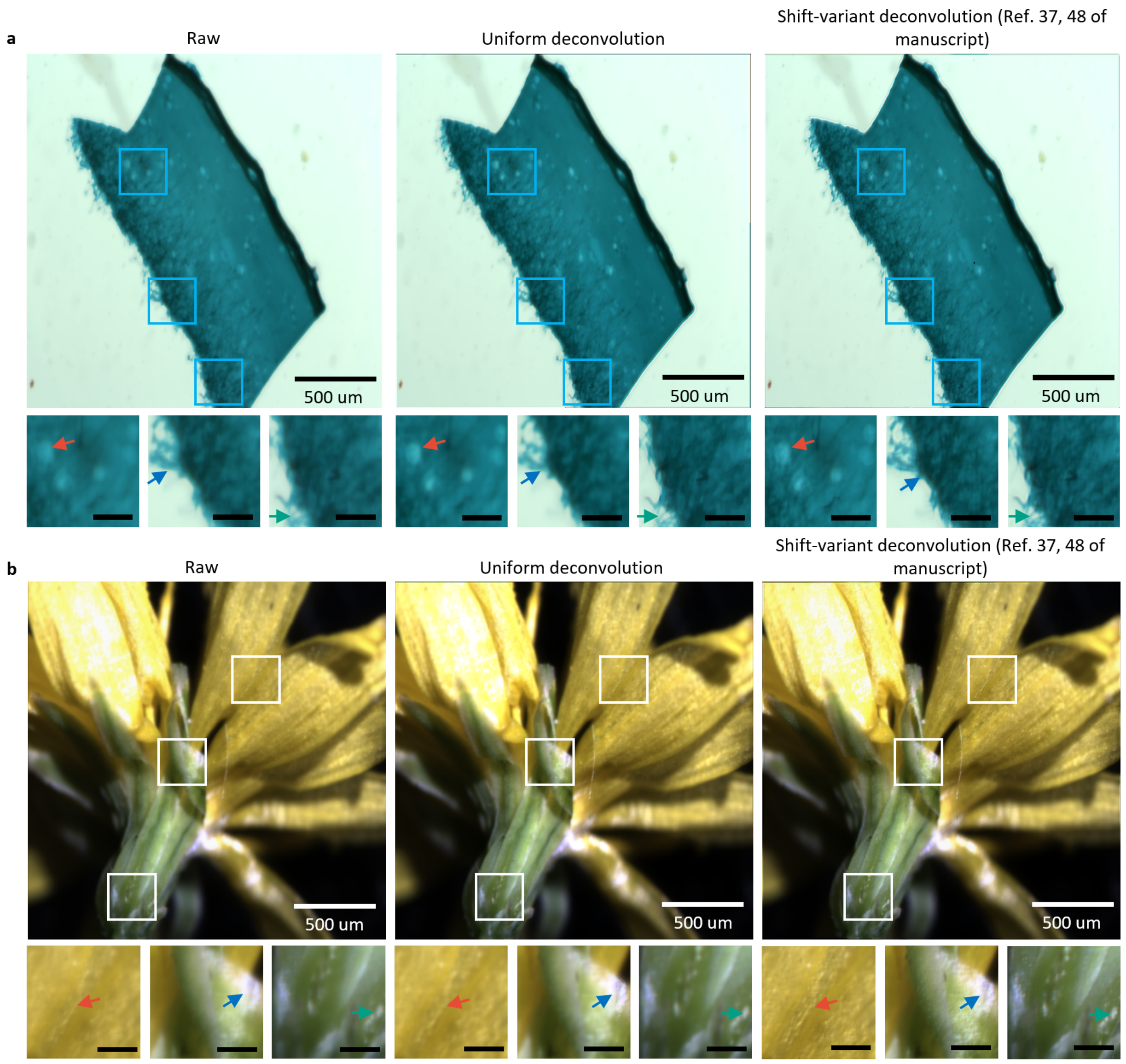

Supplementary Fig. 6. Shift-variant deconvolution algorithm outperforms uniform deconvolution algorithm. **a, b**. Two kinds of samples (Penicillium and flower, respectively) are shown to demonstrate performance comparisons of restored images obtained by uniform (middle, scale bar: 500 µm) and shift-variant deconvolution (Ref. 37, 48 of the manuscript, right, scale bar: 500 µm) from blur images (left, scale bar: 500 µm). Zoom-in areas are listed in the bottom for further comparisons (scale bar: 100 µm), and red, blue, and green arrows show three typical features (scale bar: 100 µm), where shift-variant deconvolution achieves clearer restorations than uniform deconvolution. Representative data from 122 samples.

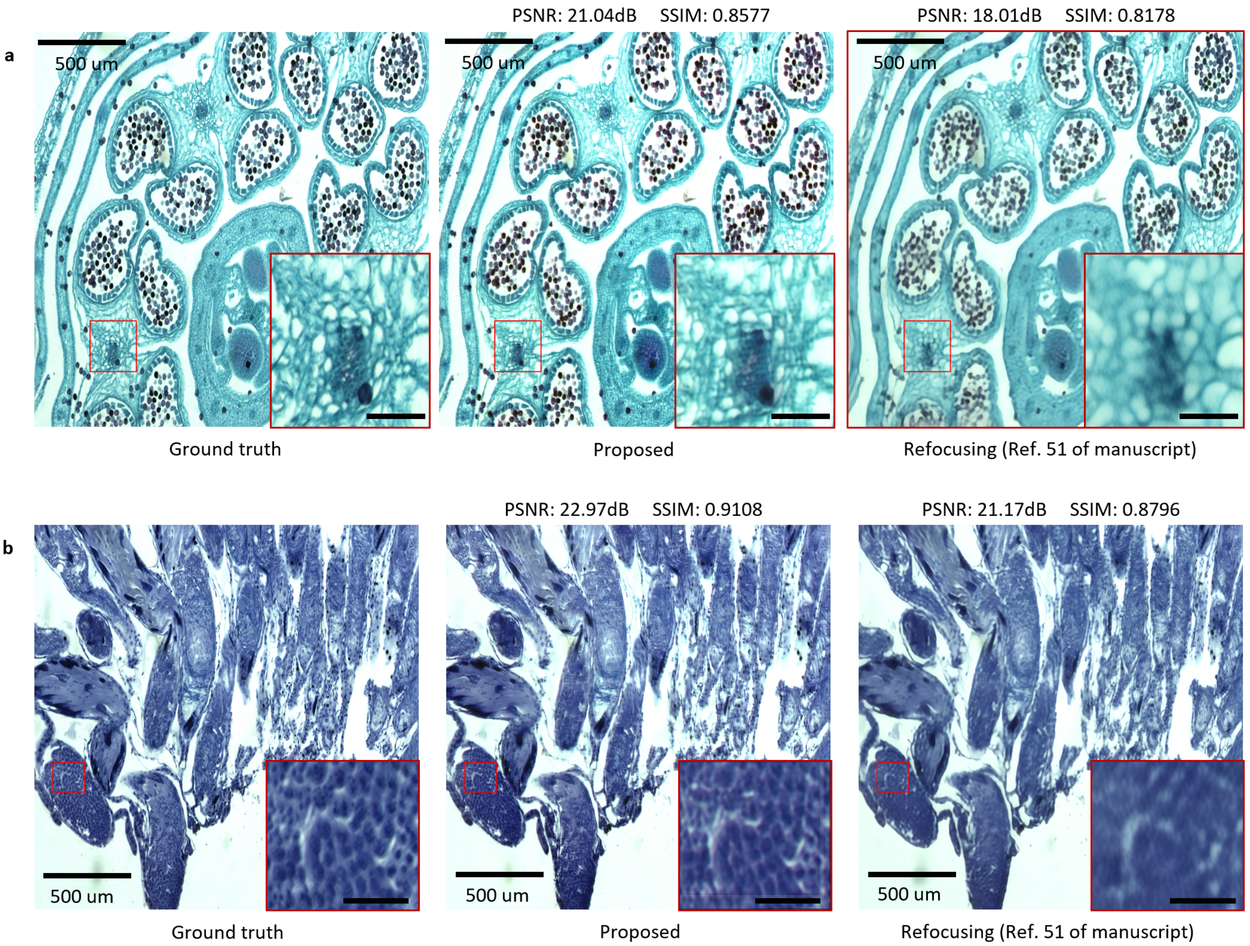

Supplementary Fig. 7. Comparisons of refocusing method with simulation-supervision method in the integrated microscope.

a, b. Two samples (rape bud and meiosis of locust testis, respectively) are presented to illustrate capability contradistinctions of recovered images acquired by simulation-supervision method (middle, scale bar: 500 μm) and refocusing method (Ref. 51 of manuscript, right, scale bar: 500 μm) compared to the ground truth (left, scale bar: 500 μm). Zoom-in areas (scale bar: 100 μm) are listed in the bottom right of each complete image, where proposed simulation-supervision maintains sharper restorations and more effective image quality metrics (peak signal-to-noise ratio, PSNR; structural similarity index, SSIM) than refocusing method. Representative data from 122 samples.

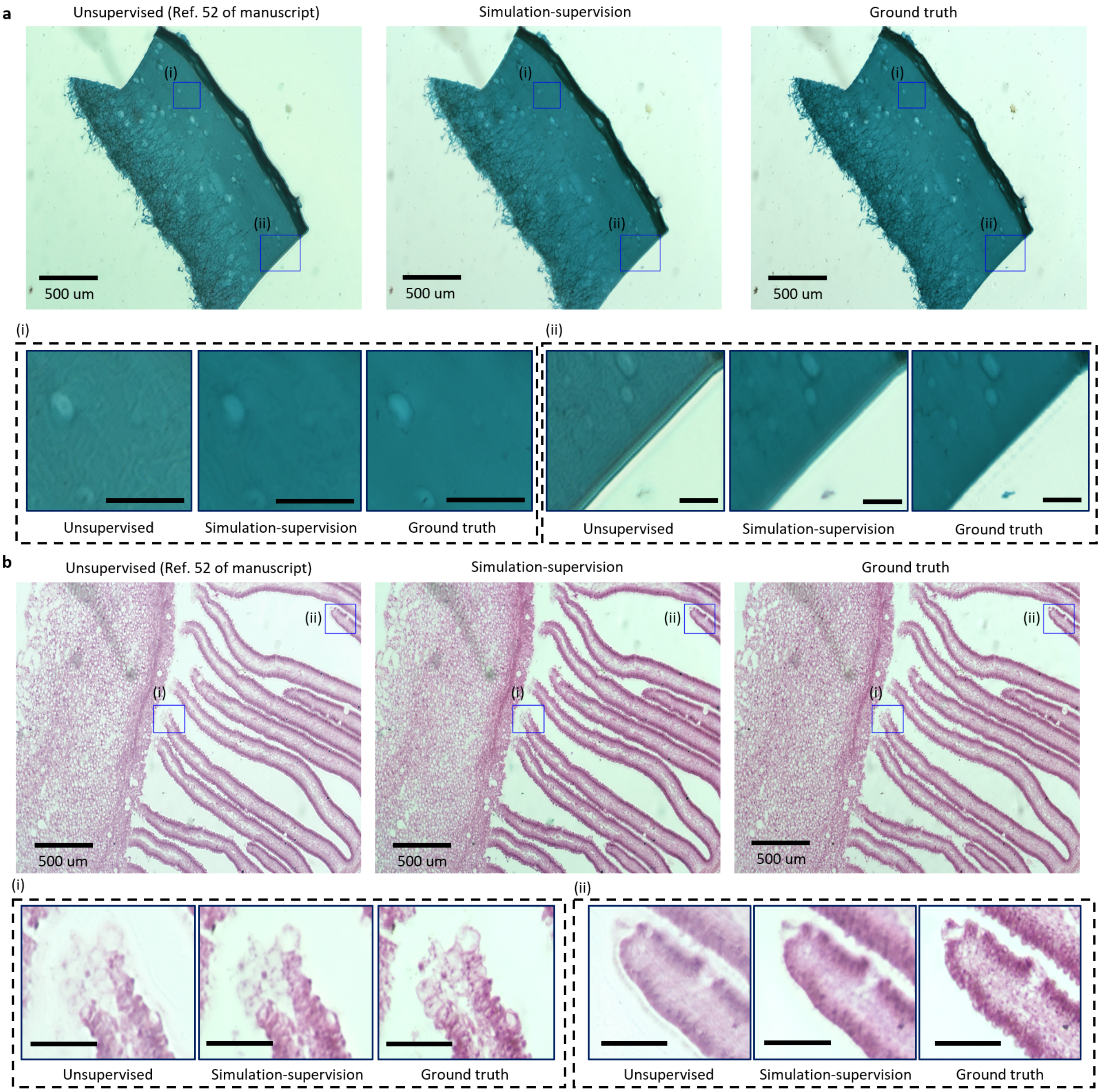

Supplementary Fig. 8. Comparisons of unsupervised method with simulation-supervision method in the integrated microscope.

a, **b**. Restoration of test samples (Penicillium and Agaricus, respectively). We test a cycleGAN model (Ref. 52 of manuscript) which does not need paired training data. On the basis of the ground truth (right, scale bar: 500 μm) we find our simulation-supervision approach (middle, scale bar: 500 μm) has higher fidelity and less boundary artifacts compared to unsupervised approach (left, scale bar: 500 μm). Two sub-characteristics (i) and (ii) are extracted to be magnified and listed inside the dashed box below for further comparisons (scale bar: 100 μm). Representative data from 122 samples.

c. Statistical comparisons of the restoration quality between the unsupervised method and proposed simulation-supervised network on 19 samples in terms of peak signal-to-noise ratio (PSNR, left), structural similarity index (SSIM, middle), and perceptual loss (Learned Perceptual Image Patch Similarity, LPIPS, right). Central line inside the box: Median. Box: interquartile range. Whiskers: Maximum and minimum. Outliers: Individual data points.

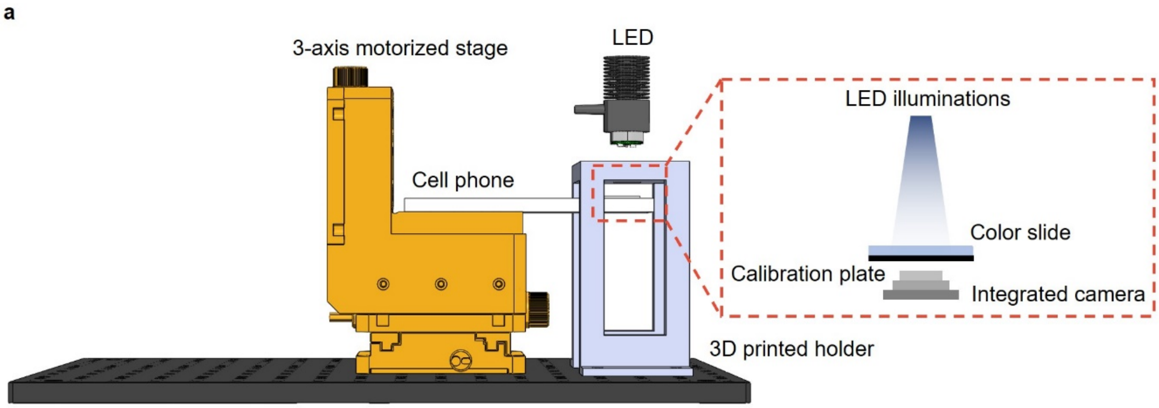

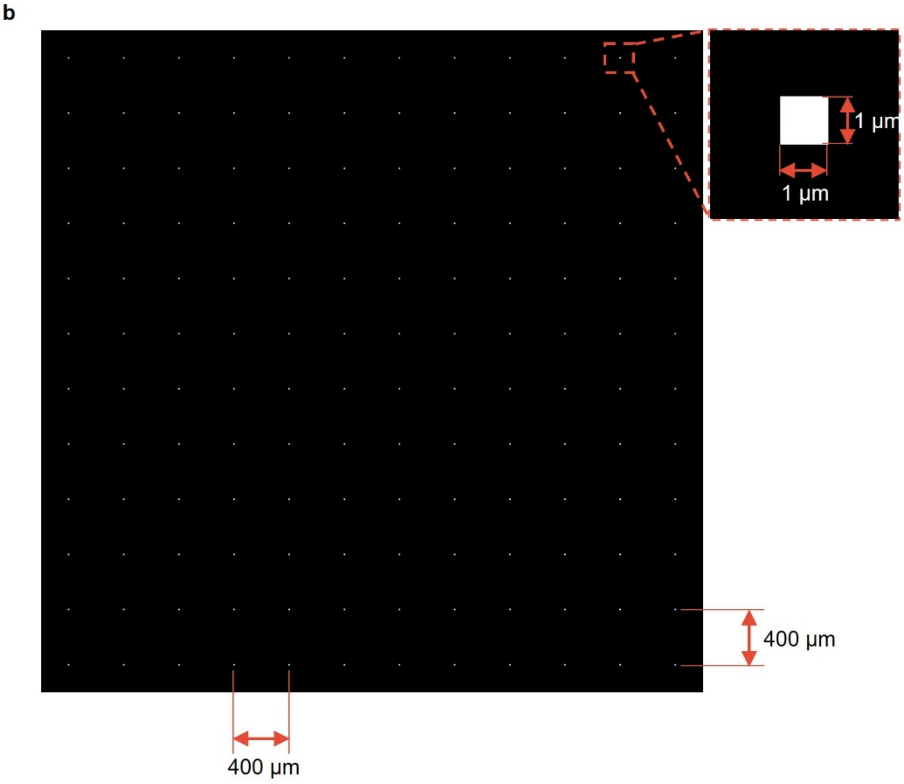

Supplementary Fig. 9. Spatially nonuniform PSF calibrations across a large FOV of the integrated microscope.

a. A sketch of point spread function (PSF) calibration setup, which consists of a 3-axis motorized stage, a LED source, a 3D printed holder, and an integration camera (assembled in a cell phone). The red dashed box illustrates that the LED illumination beams are firstly uniformed by a plastic color slide, then pass through the dot array and collected by the integrated camera. The motorized stage can carry the integrated camera to realize 3D calibrations.

b. A sketch of fabricated dot array by lithography. The separation distance between adjacent dots is 400 μm, and each dot is 1 x 1 μm^2. The black part will block the light and the white part is transparent.

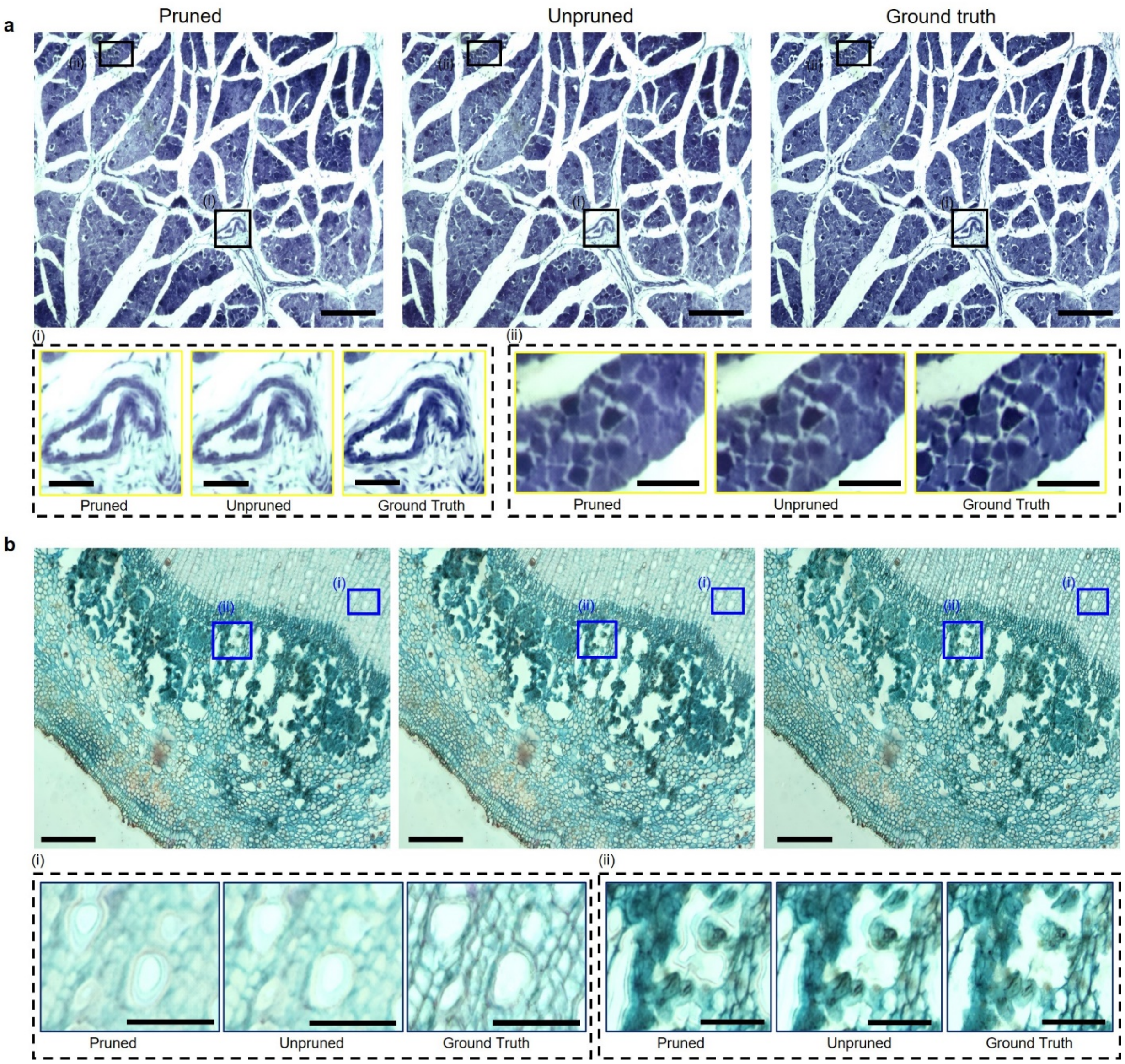

Supplementary Fig. 10. Pruned network has comparable fidelity to unpruned network with reduced computational costs.

a, **b**. Restoration of test samples (skeletal muscle and oleander stem, respectively) with pruned neural network (left, scale bar: 500 µm) and unpruned neural network (middle, scale bar: 500 µm). We find pruned neural network keeps high fidelity compared to unpruned approach in accordance with the ground truth (right, scale bar: 500 µm). Two sub-features (i) and (ii) are extracted to be enlarged and listed inside the dashed box below for further comparisons (scale bar: 100 µm). Representative data from 122 samples.

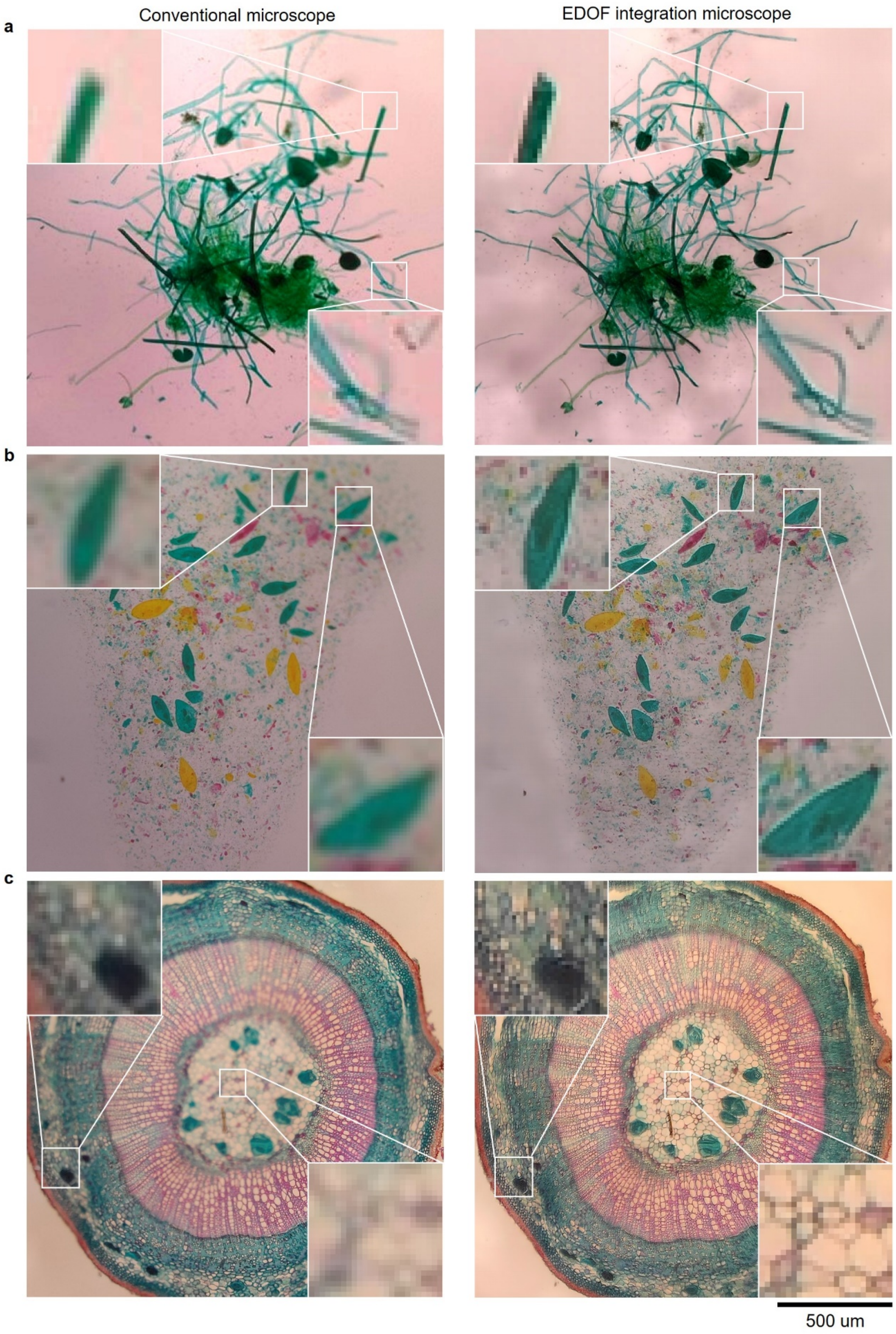
Conventional microscope
EDOF integration microscope
a
b
c
500 um

Supplementary Fig. 11. Comparisons of conventional microscope and the proposed integrated microscope equipped on a cell phone.

a, **b**, **c.** are restorations of Rhizopus nigricans, Paramecia, and root samples, respectively. Those slices are tilted to show that the proposed integrated microscope (right, scale bar: 500 μm) achieves better resolution than the conventional microscope (left, scale bar: 500 μm) in defocus areas. The zoom-in insets show the finer structural information difference of samples between two microscopes. Representative data from 106 samples.

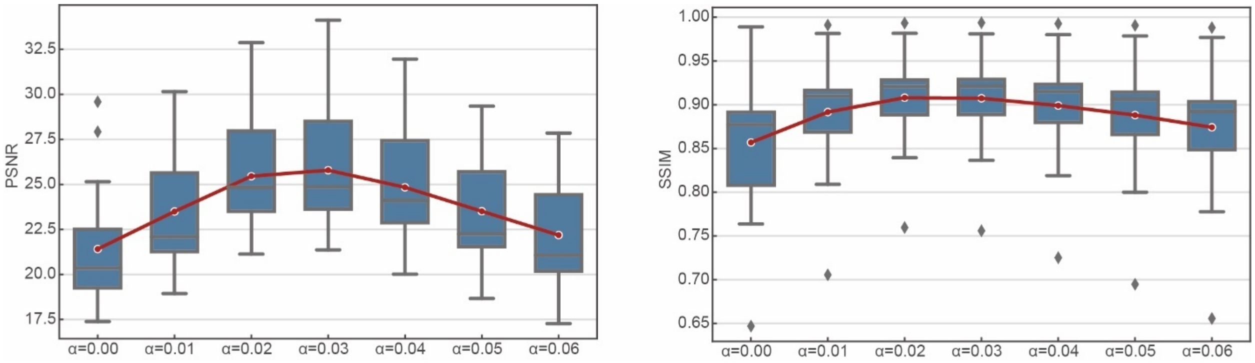

Supplementary Fig. 12. Comparison of the final output of the integrated microscope scales with different coefficients (α) of the cubic phase plate (CPP).

The left panel is peak signal-to-noise ratio (PSNR) and the right panel is structural similarity index (SSIM). Central line inside the box: Median. Box: interquartile range. Whiskers: Maximum and minimum. Outliers: Individual data points. Red lines connected mean values. n = 19 samples for each configuration.

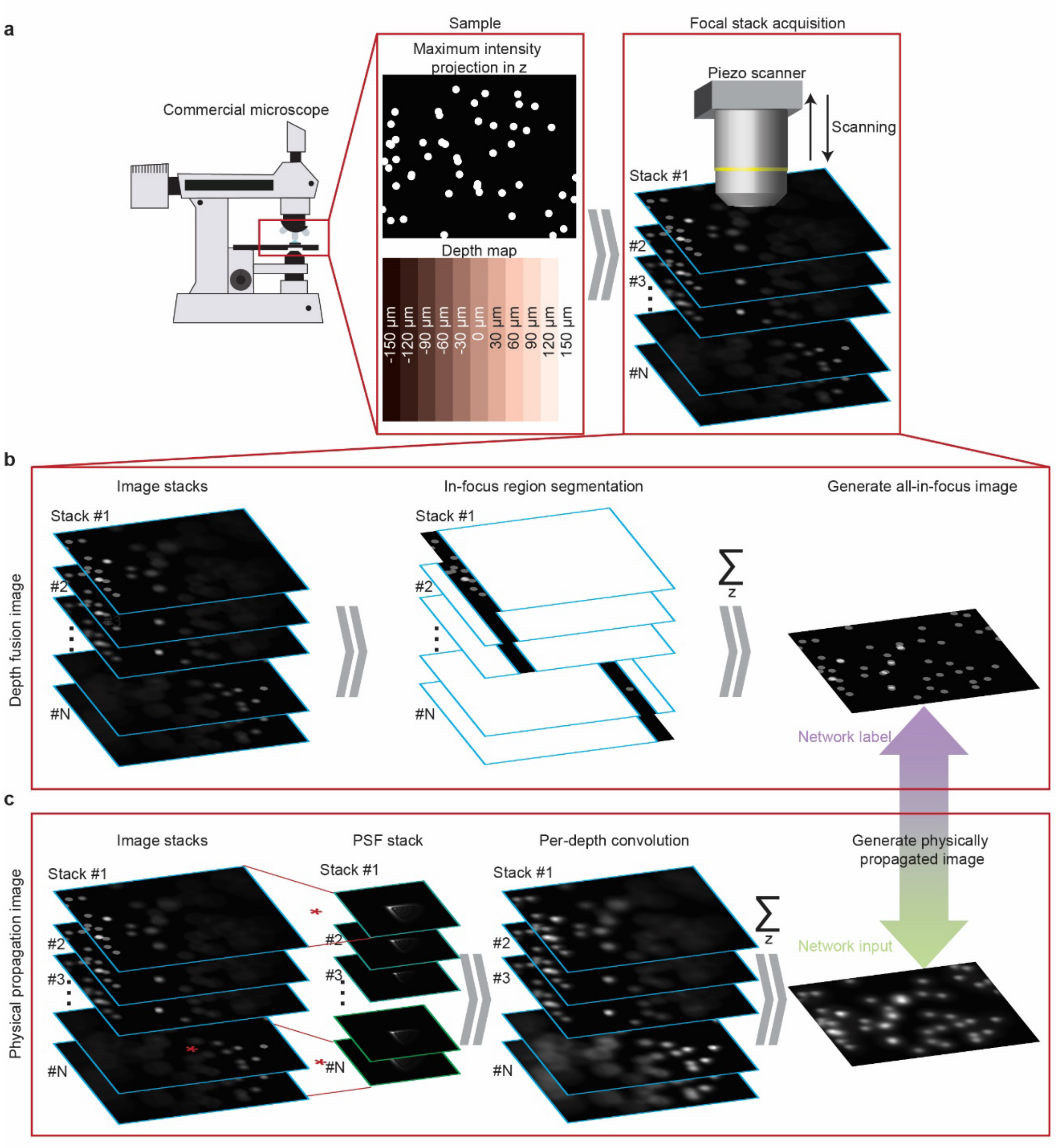

Supplementary Fig. 13. Illustrations of generating training pairs through the simulation supervision strategy.

a. Acquiring focal stacks as preparation to create training pairs for the integrated microscope. We employed a commercial microscope (left) and virtually scanned the objective to acquire a focal stack (right). As the purpose of clearer illustration, here we simulated a sample with fluorescent beads situated in 10 distinct depths (middle).

b. Generation of all-in-focus images as the network label through depth fusion. Each slice from the captured focal stack (left) was first processed to extract the region where the sample was clearly captured (i.e. the in-focus region, middle). Then, these in-focus regions were summed to create an all-in-focus image, which we refer to as the depth-fusion image (right).

c. Generation of the simulated captured image by the integrated microscope image as the network input through physical propagation. Each slice from the captured focal stack (left) was firstly convolved with the depth-specific

point spread functions (PSFs, middle), and then summed to reassemble the capture from the integrated microscope (right).

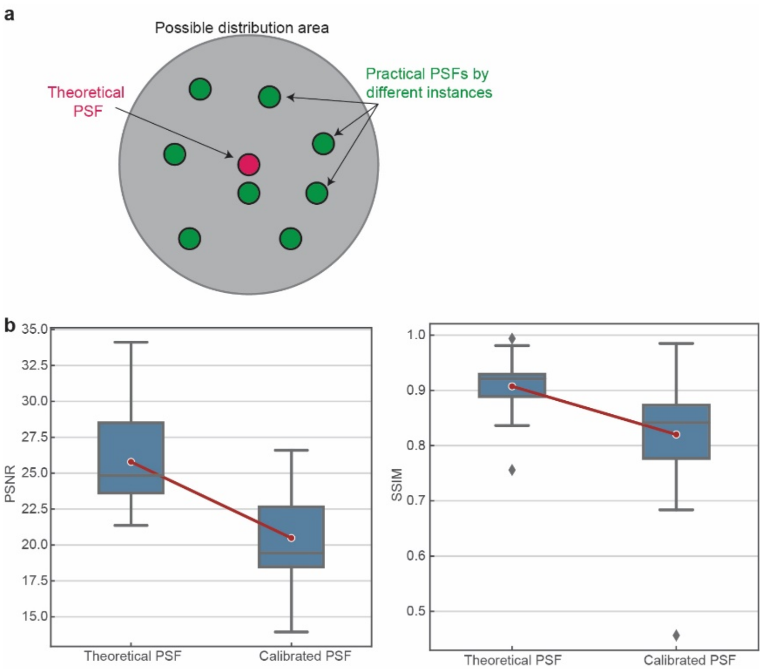

Supplementary Fig. 14. Comparison of imaging restoration results by the theoretical PSF-driven neural network and the calibrated PSF-driven neural network.

a. Conceptual illustration of the distribution of theoretical point spread function (PSF, red dot) and practical PSFs by different fabricated instances (green dots) in a high dimensional space. If we assume the fabrication flaws are distributed in a centered manner (gray disks), the theoretical PSF locates in the center and has the closest similarity between various practical PSFs in average.

b. Comparison of imaging restoration results by neural networks driven by theoretical PSF and calibrated PSF. The left panel displays peak signal-to-noise ratio (PSNR) values (in dB) and the right panel showcases structural similarity index (SSIM) metrics. Central line inside the box: Median. Box: interquartile range. Whiskers: Maximum and minimum. Outliers: Individual data points. n = 19 samples.

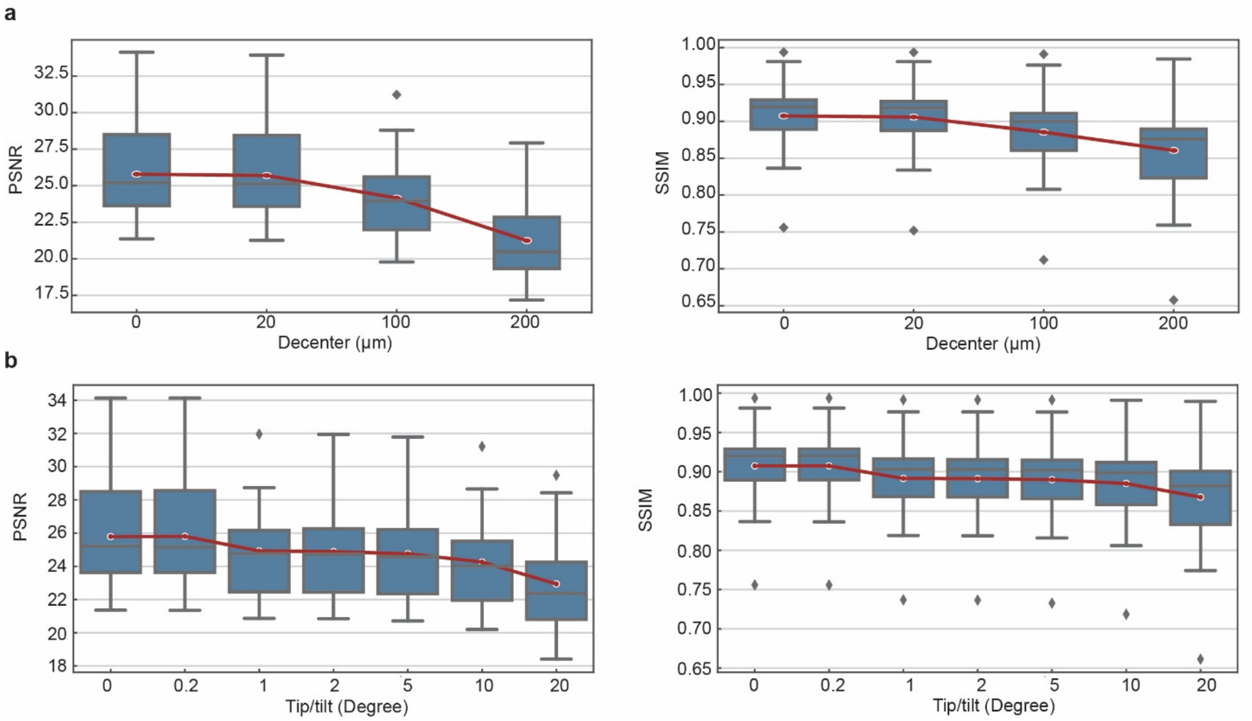

Supplementary Fig. 15. Comparison of imaging restoration results by the theoretical PSF-driven neural network across various instances of the proposed integrated microscope.

a. Evaluation of the restoration results over various instances of the integrated microscope, each featuring distinct assembly decenter discrepancies. The left panel displays peak signal-to-noise ratio (PSNR) values (in dB) and the right panel showcases structural similarity index (SSIM) metrics. Central line inside the box: Median. Box: interquartile range. Whiskers: Maximum and minimum. Outliers: Individual data points. n = 19 samples for each configuration.

b. Evaluation of the restoration results over various instances of the integrated microscope, each featuring distinct assembly tip/tilt discrepancies. The left panel displays PSNR values (in dB) and the right panel showcases SSIM metrics. Box plot symbols as in **a**. n = 19 samples for each configuration.

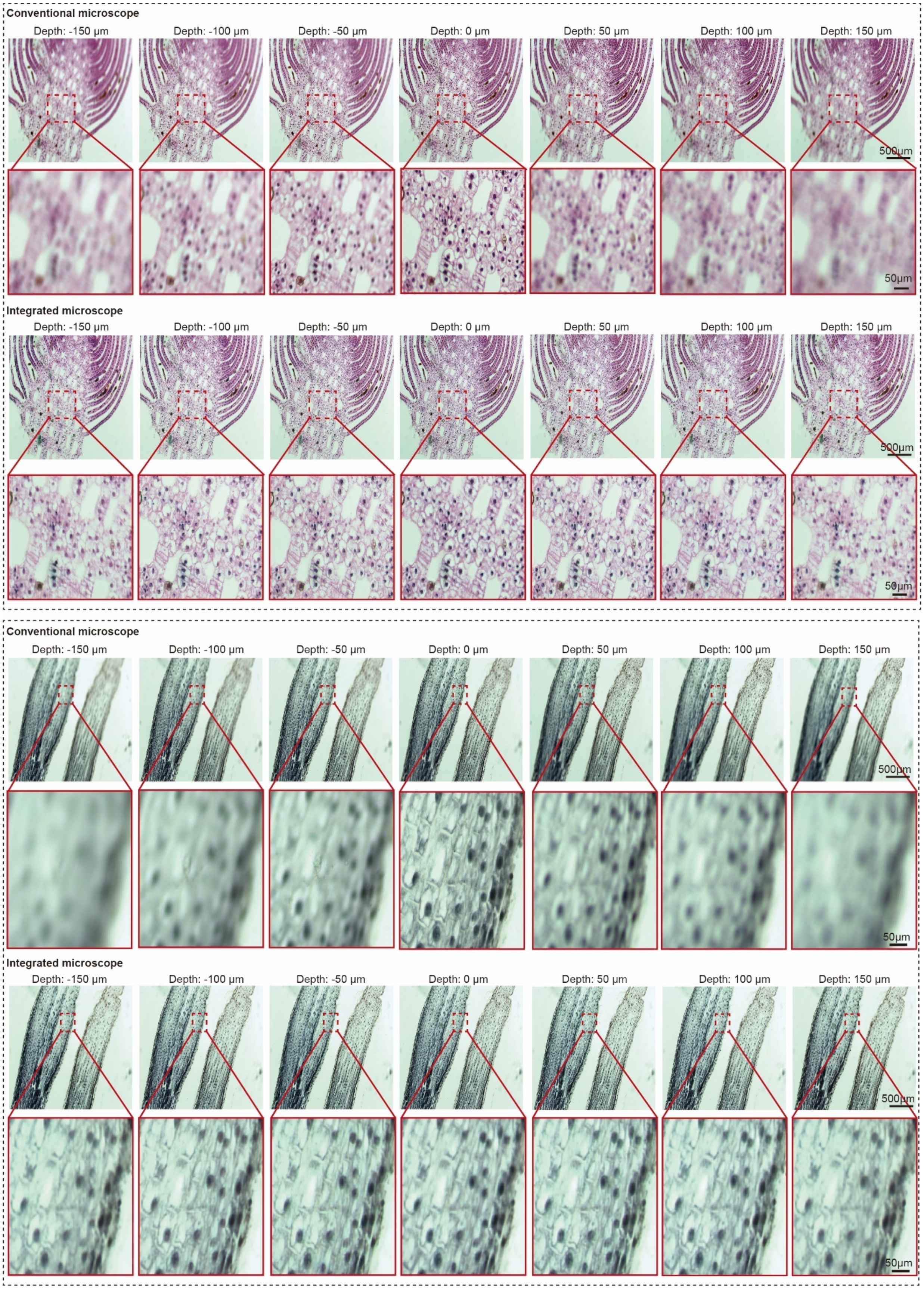
Conventional microscope
Depth: -150 µm
Depth: -100 µm
Depth: -50 µm
Depth: 0 µm
Depth: 50 µm
Depth: 100 µm
Depth: 150 µm
500µm
50µm
Integrated microscope
Depth: -150 µm
Depth: -100 µm
Depth: -50 µm
Depth: 0 µm
Depth: 50 µm
Depth: 100 µm
Depth: 150 µm
500µm
50µm
Conventional microscope
Depth: -150 µm
Depth: -100 µm
Depth: -50 µm
Depth: 0 µm
Depth: 50 µm
Depth: 100 µm
Depth: 150 µm
500µm
50µm
Integrated microscope
Depth: -150 µm
Depth: -100 µm
Depth: -50 µm
Depth: 0 µm
Depth: 50 µm
Depth: 100 µm
Depth: 150 µm
500µm
50µm

Supplementary Fig. 16. Comparison of imaging results of biological samples by the proposed microscope and a traditional microscope.

Two samples at different depths were recorded by both traditional microscope and the proposed integrated microscope. The red box labelled region is zoomed and exhibited in the bottom right corner in each panel. Representative data from 19 samples.

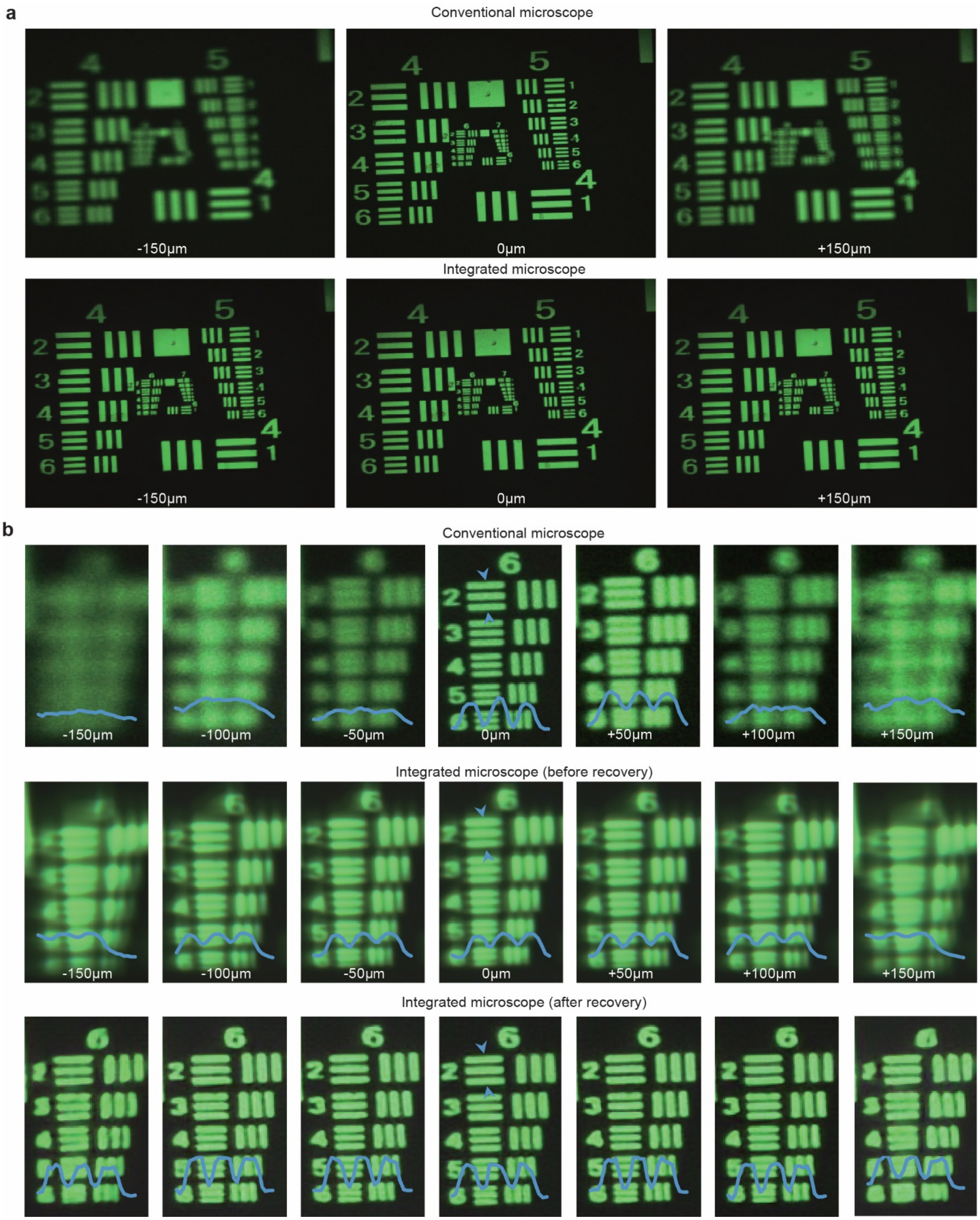

Supplementary Fig. 17. Comparison of imaging results of a USAF1951 resolution chart through conventional microscope and integrated microscope in detail.

a. Imaging results with a conventional microscope from -150 μm to +150 μm defocus range. Top was by a conventional microscope that has the similar optical parameters but without cubic phase plate (CPP) coding. Bottom was by the proposed integrated microscopes.

b. Zoom-in view of the USAF1951 resolution target in **a** at different depths. The intensity profiles across group 6, element 2 of the resolution targets are plotted in blue curves in the bottom. The first row was by the conventional microscope, the second row was by the proposed integrated microscopes without applying the recovery network, and the third row was by the proposed integrated microscopes with applying the recovery network.

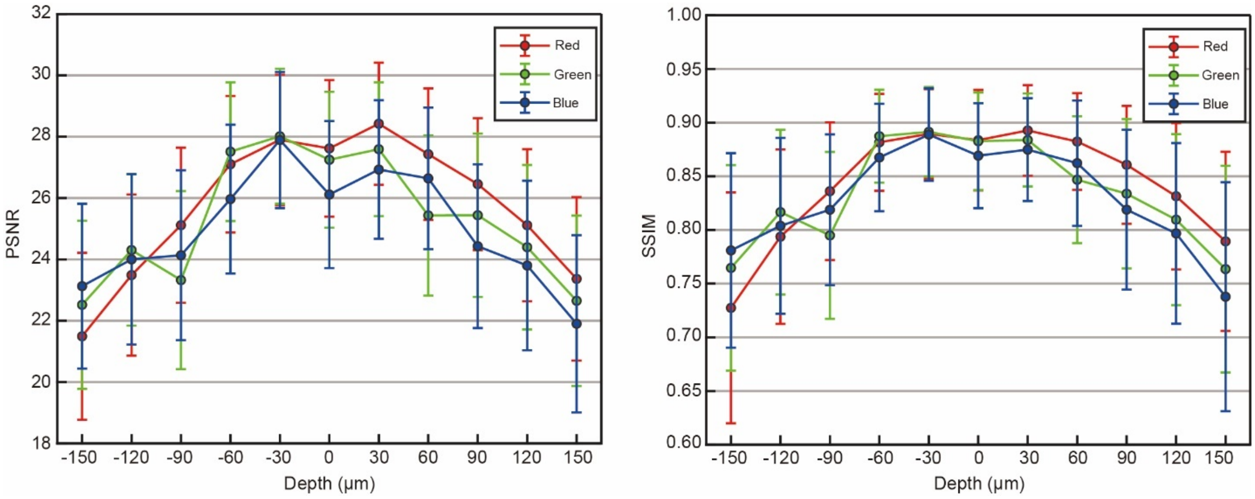

Supplementary Fig. 18. Assessment of the impact of chromatic aberration on the extension of depth.

For each detection channel of the sensor (red, green, and blue), we assessed the axial extent of the depth extension by directly applying the trained restoration neural network and evaluated the recovery results in each depth and each channel. The left panel is peak signal-to-noise ratio (PSNR) and the right panel is structural similarity index (SSIM). n = 19 samples per depth. Height of circles: Mean. Error bars: SD.

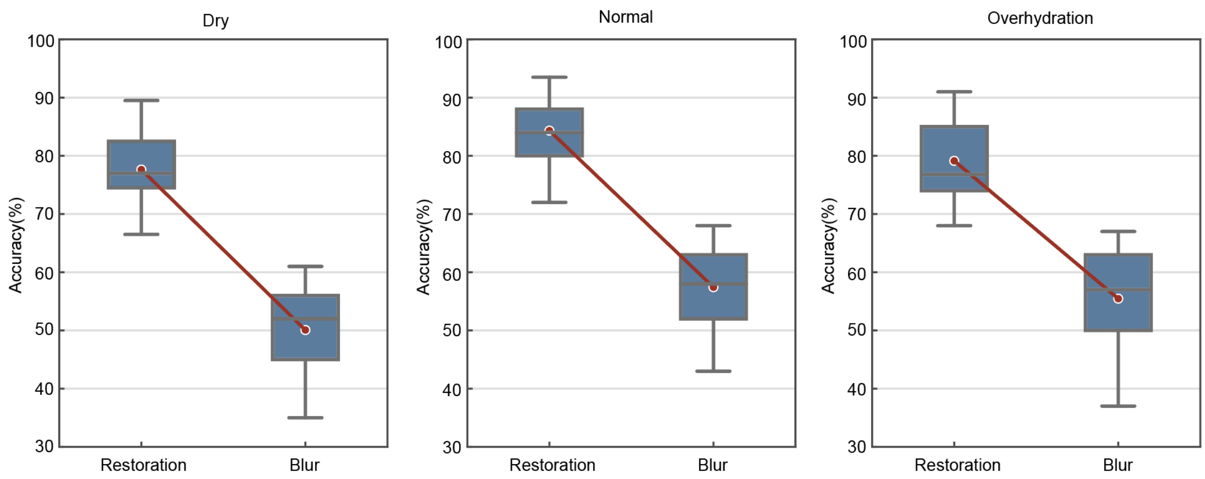

Supplementary Fig. 19. Ablation study of skin state diagnosis related to the resolution of the skin images.

We trained two identical diagnostic neural networks to classify skin images into dry (left), normal (middle), and overhydrated (right) categories, using either raw captures (Blur) or restored high-resolution images (Restoration). The classification accuracies are visualized as box plots. Central line inside the box: Median. Box: interquartile range. Whiskers: Maximum and minimum. Outliers: Individual data points. Red lines connected mean values. n = 28 samples.

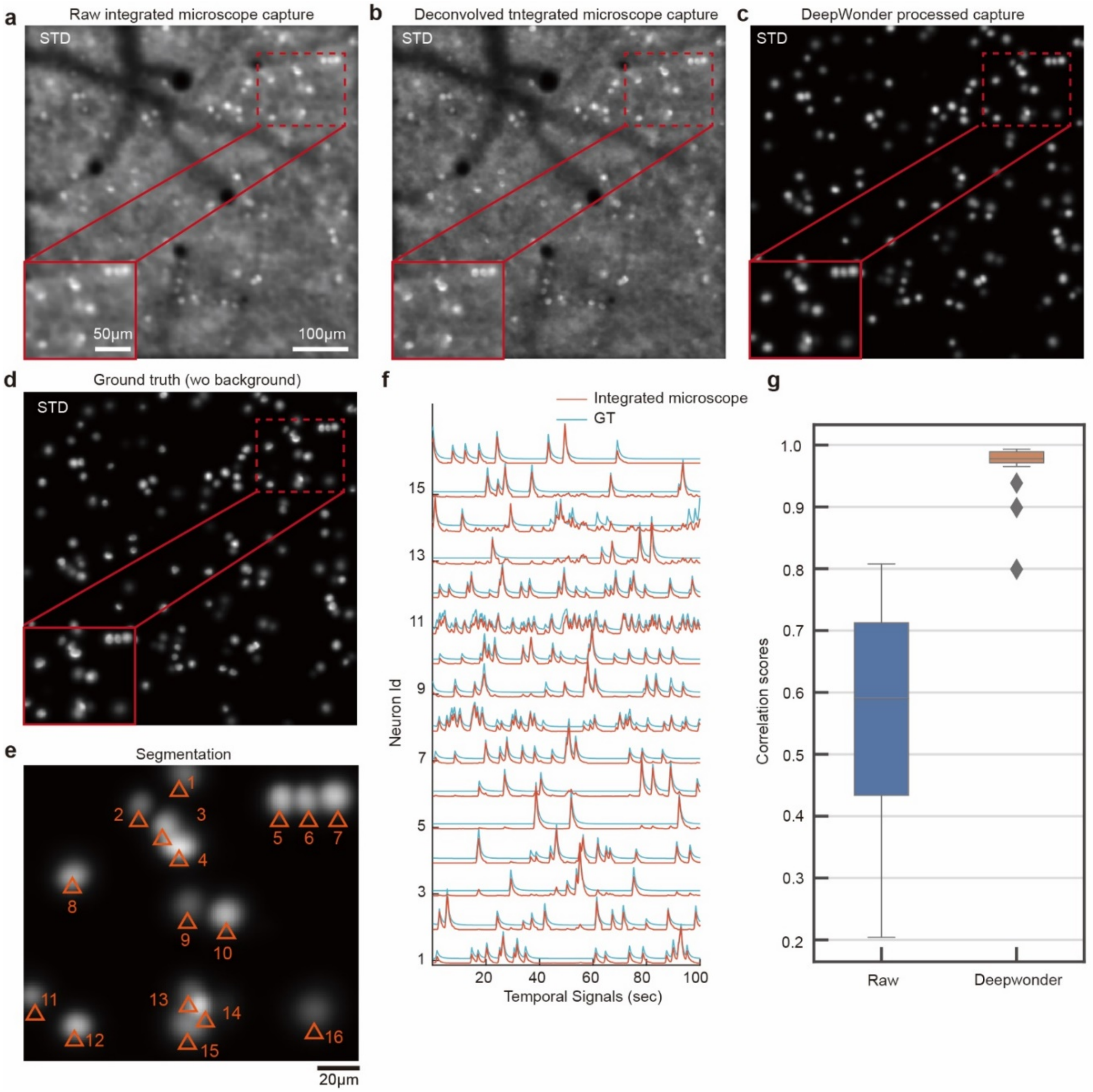

Supplementary Fig. 20. Simulation of the integrated microscope in capturing cortical neuron activities.

We combined the NAOMi simulator [1] with the optical model of the proposed integrated microscope to generate virtual calcium recordings to evaluate the calcium activity recording ability of the proposed integrated microscope.

a. Standard deviation (STD) image across temporal frames of raw movie captured by the proposed integrated microscope.

b. STD image across temporal frames of deconvolved movie captured by the proposed integrated microscope.

c. DeepWonder[2] processed movie of the proposed integrated microscope. Typically, we first deconvolved the captured cortical image using the proposed recovery neural network. Subsequently, we employed the DeepWonder algorithm to eliminate background contamination.

d. Simulated ground truth (GT) movie (without background contamination), as reference.

e. Segmented integrated microscope captures. Each segmented neuron was marked by a triangle and was numbered.

f. Neuronal activity traces as used for performance quantifications. Neurons that were numbered in **e** were collected and ordered. Red traces for DeepWonder movie and blue traces for ground truth movie, and DeepWonder traces are offset vertically for clarity.

g. Temporal correlations of detected neurons with ground truth from DeepWonder output movie (0.96 ± 0.57, mean ± SD, blue) and raw movie (0.57 ± 0.18, mean ± SD, red), across n = 16 neurons. Central black mark: Median. Bottom and top edges: 25th and 75th percentiles. Whiskers extend to extreme points excluding outliers (1.5 times above or below the interquartile range).

Supplementary Table 1. Comparisons of the proposed integrated microscope with other miniaturized microscopes.

The comparisons include weight, volume, resolution, the field of view, and depth of field.

	Miniscope v3	Miniscope v4	MiniLFOV	Miniscope 3D	Minifast	Mesoscope	Featherscope	Ours
Weight	3.2 gram	2.6 gram	13.9 gram	2.5 gram	3.45 gram	4.5 gram	1.0 gram	0.5 gram
Volume	~17mm x13mm x23mm	~13mm x 13mm x 22mm	~35mm x 20mm x 35mm	~17 mm x13mm x23mm	~17mm x13mm x23mm	~17 mm x 36 mm x 9 mm	~6mm x 15mm x 15mm	~5mm x 5mm x 6mm
Resolution	~1.6 µm	~2 µm	~3 µm	2.76 µm	1.5 µm	~ 5 µm	~4 µm	3 µm
Field of view	0.7mm x0.5mm	~ Φ1 mm	3.1mm x2.3mm	~0.9mm x0.7mm	0.7mm x0.5mm	~4 mm x 3 mm	~1.0 x 1.0 mm	Φ3.6 mm
Depth of field	~15 µm	~25 µm	~25 µm	~390 µm	~15 µm	~40 µm	~40 µm	~300 µm
Reference	Ref. 27 of manuscript	[3]	Ref. 36 of manuscript	Ref. 37 of manuscript	Ref. 38 of manuscript	Ref. 39 of manuscript	Ref. 40 of manuscript	

Supplementary Table 2. Full prescription data of the proposed integrated microscope.

Type	Radius	Thickness	Glass	Semi-diameter	Surface parameters
Standard	Infinity	0.300	EP-9000	0.566	
Extended polynomial	Infinity	0.120		0.447	Norm radius: 1.000<br>X3Y0: 0.030<br>X0Y3: 0.030
Standard (Stop)	Infinity	-0.020		0.348	
Even asphere	1.159	0.334	ZEONEX_K22R& K26R_2017	0.349	4th order: -0.154<br>6th order: 0.609<br>8th order: -190.180<br>10th order: 3984.058<br>12th order: -4.469E+04<br>14th order: 2.500E+05<br>16th order: -5.709E+05
Even asphere	-1.503	0.122		0.401	4th order: -2.588<br>6th order: 11.561<br>8th order: -93.332<br>10th order: 407.060<br>12th order: -2738.190<br>14th order: 1.621E+04<br>16th order: -3.887E+04
Even asphere	-2.043	0.362	EP-9000	0.425	4th order: -2.226<br>6th order: 12.109<br>8th order: -131.037<br>10th order: 830.923<br>12th order: -2926.059<br>14th order: 7570.232<br>16th order: -1.089E+04
Even asphere	-62.219	0.207		0.574	4th order: -0.753<br>6th order: 2.469<br>8th order: -9.494<br>10th order: 40.041<br>12th order: -167.800<br>14th order: 489.624<br>16th order: -527.432
Even asphere	-2.485	0.549	ZEONEX_K22R& K26R_2017	0.675	4th order: -1.130<br>6th order: 4.629<br>8th order: -17.808<br>10th order: 58.248<br>12th order: -133.519<br>14th order: 200.192<br>16th order: -135.291
Even asphere	-0.414	0.030		0.781	4th order: -1.389<br>6th order: 5.524<br>8th order: -18.141<br>10th order: 40.662<br>12th order: -50.310<br>14th order: 37.270<br>16th order: -14.720

Even asphere	2.334	0.432	ZEONEX_K22R&K26R_2017	1.168	4th order: -0.143<br>6th order: -0.699<br>8th order: 2.040<br>10th order: -2.769<br>12th order: 2.030<br>14th order: -0.767<br>16th order: 0.114
Even asphere	0.364	0.690		1.532	4th order: -0.266<br>6th order: 0.281<br>8th order: -0.215<br>10th order: 0.104<br>12th order: -0.032<br>14th order: 6.048E-03<br>16th order: -5.759E-04
Standard	Infinity	0.210	BK7	1.694	
Standard	Infinity	0.333		1.770	
Standard (Image)	Infinity			1.971	

Supplementary Note 1: Reproducible workflow of the proposed design approach

To validate the capability of the proposed design approach in designing arbitrary lenses, here we introduced another case as a demonstration to assist readers in applying our design approach to various systems.

The demo starts with a simple 10x microscopic system with NA 0.24 (Supplementary Fig. 21), and aims to use diffractive optical elements (DOEs) to extend the depth of the field (DOF) of the system. In the first step, we optimized the 10x microscopic system directly through ray tracing, with the merit of high MTFs values across the 2 mm field-of-view (FOV), and equivalized MTF across a wide wavelength range (470-650 nm). The adaptive gradient descent algorithm was applied to optimize the surface shape to reduce aberrations. As a result, the system exhibits a sharp focus near the focal plane, but the point spread function (PSF) largely spreads in defocused planes (Supplementary Fig. 21c, 21d).

In the second step, we inserted a DOE with cubic phase distribution near the pupil plane of the first achromatic lens, to encode the light field and enhance the depth invariance of the PSF (Supplementary Fig. 22a). We finetuned the surface parameters of the 10x microscopic system such that MTFs of the system are similar across 300 µm DOF. We found after inserting the DOE, the MTFs across lateral positions and different defocus depths became similar (Supplementary Fig. 22b-22e). Varying different parameters of the DOE yielded different optical systems (combination of the lenses and the DOE) with varied MTFs and DOF extension ability.

Thus in the third step, we enlisted an end-to-end neural network to select the best of configuration. The neural network had the same structure we used for the proposed integrated microscope in the manuscript (Fig. 2 of the present manuscript). We separately trained neural networks for each configuration with merits of best reconstructions across 300 µm depth range. As depicted in Supplementary Fig. 23, we found that α = 40 resulted in the best imaging results based on structural similarity index (SSIM) and peak signal-to-noise ratio (PSNR) metrics, and thus this value was selected as the optimal CPP parameter.

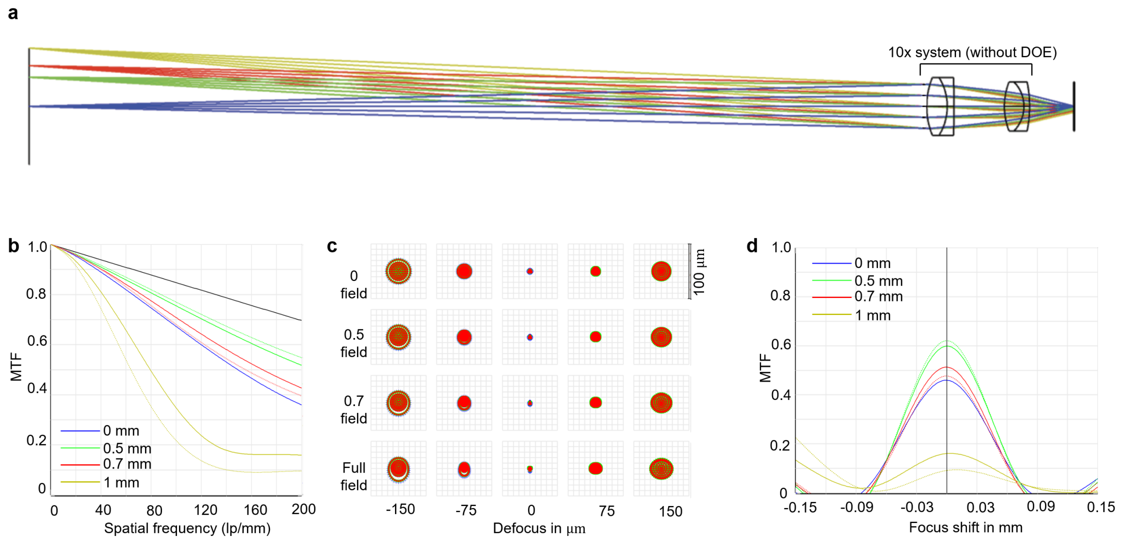

Supplementary Fig. 21. Optical characteristics of an optimized 10x microscopic system without diffractive optical elements (DOE). **a**. The 10x microscopic optical design contains two sets of achromats. The effective focal length is 15 mm and the NA is 0.24. No DOE was inserted. The system was optimized across 2 mm fields and visible wavelengths. **b**. The MTF curves across different spatial frequencies for different lateral field positions. The black solid line indicates the diffraction limit. **c**. Spot diagrams at different focal planes and lateral positions. Different color corresponds to different wavelengths. **d**. MTF at different focal planes of with a spatial frequency of 100 cycles per mm.

In the next we explained in detail of required software/hardware that is needed to replicate these results. The involved software in the above workflow includes Zemax for ray-tracing-based optimization (step 1, step 2), and deep-learning tools (like Pytorch) for neural network-based configuration selection (step 3). On the other hand, the hardware that is required for fabricating the designed systems is mostly depending on the complexity of the optical system. For the aspherical lens-based system presented in the manuscript (integrated microscope), the manufacturing process typically involves a combination of CNC machining, injection molding, and surface coating. The lens barrel was machined from a solid block of aluminum using a CNC machine. The lens elements were injection molded using specialized equipment that was designed to produce high-quality optical polymers. Once the lens elements were produced, they are assembled into the lens barrel and the entire assembly is coated with an anti-reflective coating to improve image quality. For the demo system as proposed in Supplementary Fig. 21-23, glass materials were preferred and the fabrication can be easier. The lens blank is shaped and polished using a series of grinding and polishing tools as the goal to create the desired curvature and smoothness on the surface of the lens. After that, the anti-reflection coatings were applied to the lens surfaces to reduce unwanted reflections and improve contrast.

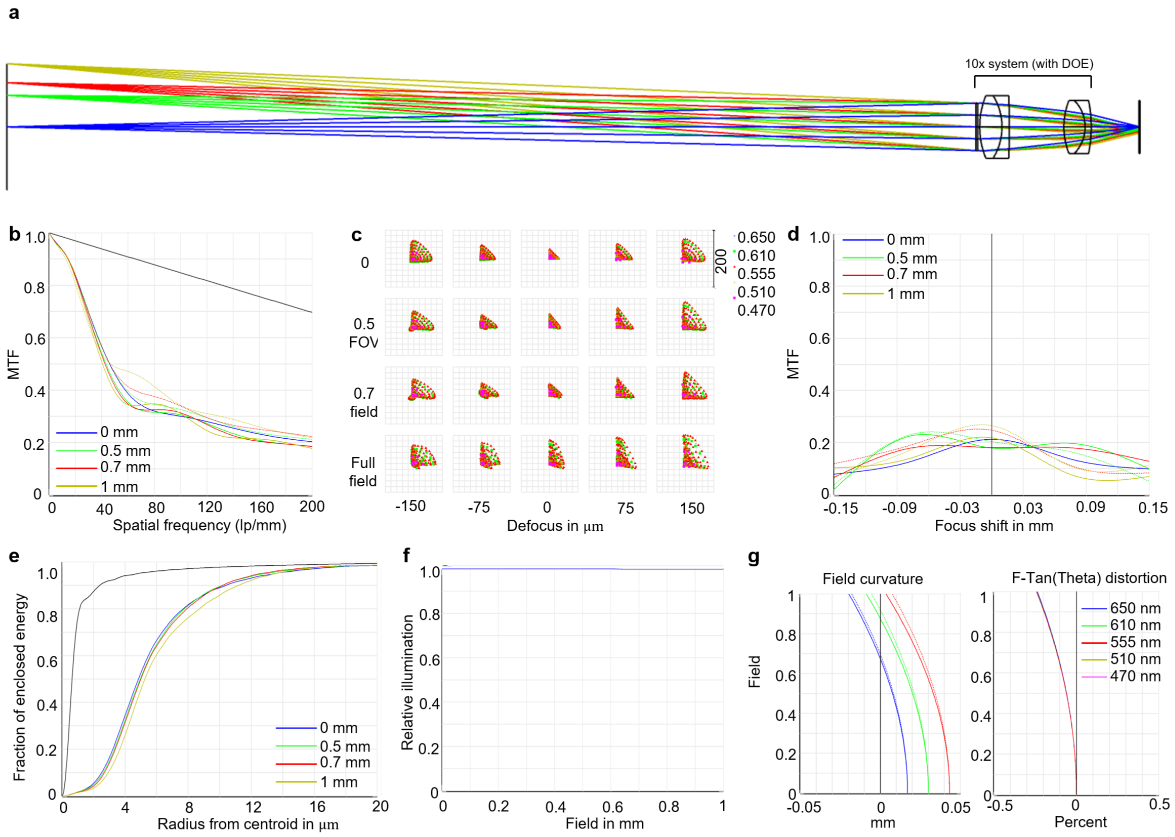

Supplementary Fig. 22. Optical characteristics of an optimized 10x microscopic system with diffractive optical elements (DOE). **a**. The 10x microscopic optical design contains two sets of achromats and a DOE for depth-of-field (DOF) extension. **b**. The MTF curves across different spatial frequencies for different lateral field positions. The black solid line indicates the diffraction limit. **c**. Spot diagrams at different focal planes and lateral positions. Different color corresponds to different wavelengths. **d**. MTF at different focal planes with a spatial frequency of 100 cycles per mm. **e**. Diffraction Encircled energy across different field radii. **f**. Relative illumination intensity across different field positions. **g**. Field curvature and distortion across different field positions of different wavelengths.

The selection of a fabrication process for DOEs is contingent upon the specific type of DOE and its intended purpose. In the case of the proposed integrated microscope discussed in our manuscript, we opted for nanoimprinting as our chosen fabrication process in order to facilitate mass production. The mold for nanoimprinting was created using two-photon polymerization with the desired nanostructure patterns. The mold is then pressed onto the substrate surface, transferring a pattern from the mold to the substrate. Following this, the imprint is cured through UV light, and the mold and residual material are subsequently removed from the substrate. Regarding the proposed demo system in Supplementary Fig. 21**-**23**,** gray-scale lithography may be utilized for fabricating DOEs for lab use and testing purposes, given that the scale of fabrication required is not particularly extensive.

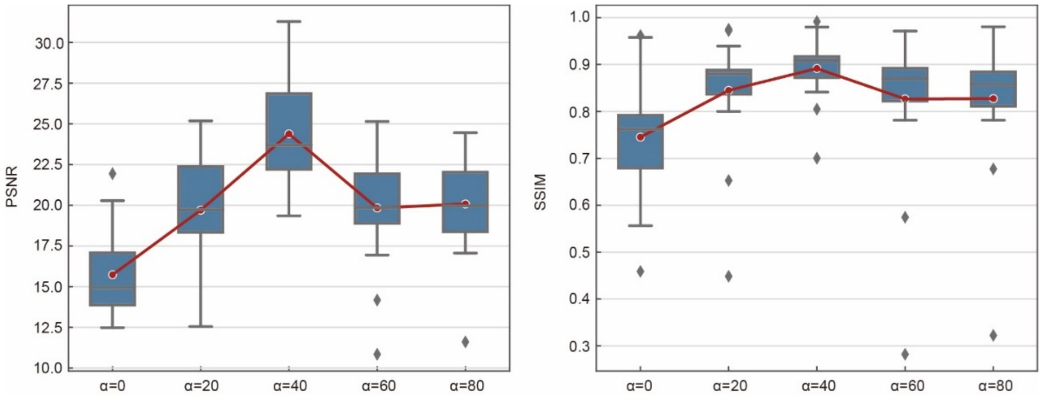

Supplementary Fig. 23. Comparison of the final output of the integrated microscope scales with different coefficients (α) of the cubic phase plate (CPP) in a 10x microscopic system. The left panel is peak signal-to-noise ratio (PSNR) and the right panel is structural similarity index (SSIM). Central line inside the box: Median. Box: interquartile range. Whiskers: Maximum and minimum. Outliers: Individual data points. Red lines connected mean values. n = 19 samples for each configuration.

The time required to follow our proposed design pipeline is typically several days to a week long. Step 1 and Step 2 can be completed quickly if the designer is familiar with the ray-tracing software. Step 3 entails running a neural network multiple times to select the optimal configuration. Refining Step 1 and Step 2 took a week in the system proposed in the manuscript, while training multiple neural networks in Step 3 took a total of 100 hours. The duration of the fabrication process can vary significantly based on the complexity of the procedures involved and the accessibility of the necessary machinery.

Supplementary Note 2: Comparison of the proposed integrated microscope with Foldscope and Cellscope

Based on the public parameters, here we compare our proposed integrated microscope with Manu Prakash's Foldscope and Cellscope side-by-side.

Device	Integrated microscope (proposed)	Manu Prakash's Foldscope	Cellscope (with 10x objective)
EFL	1.15 mm	1.76 mm	Unknown
BFL	0.75 mm	0.56 mm	Unknown
NA	0.16	0.2	0.25
FOV	Φ1.89 mm	Φ0.52 mm	Φ0.80 mm
DOF	300 µm	13.7 µm	17.45 µm
Resolution	2.5 µm	1.9 µm	1.7 µm
Size	~ 6.5 mm x 6.5 mm x 4 mm	~ 11.3 cm x 3.9 cm x 1.8 cm	~ 144 mm x 45 mm x 133mm
Cost	<10 USD (under mass production, sensor included)	~10 USD (including a sensor)	>200 USD (including objective and eyepiece)

Supplementary Table 3. Comparisons of the proposed integrated microscope with other cellphone-based Foldscope [4] and Cellscope [5]. The comparisons include effective focal length (EFL), back focal length (BFL), numerical aperture (NA), the field-of-view (FOV), depth-of-the-field (DOF), optical resolution, size, and cost.

The resolutions of the three devices are comparable, yet their field of view (FOV) and depth of field (DOF) differ significantly. The Foldscope employs a spherical ball lens as its primary objective, which introduces considerable field-dependent aberrations, akin to the Cellscope which lacks joint optimization between microscopic beam paths and phone lenses. Conversely, the integrated microscope features four aspherical lenses and cubic phase plates, which are jointly optimized, resulting in an effective FOV 13 times larger than the Foldscope and 5.6 times larger than the Cellscope. Furthermore, the integrated microscope incorporates a cubic phase plate to extend its depth of field, enhancing image quality under handheld conditions. In contrast, both the Foldscope and Cellscope lack such a component and are susceptible to axial drift.

The costs of the integrated microscope and Folscope are comparable, while the cost of Cellscope is significantly higher since the employment of commercial objective and eyepiece. On the other hand, the usability of these three varies. Recording with the Foldscope necessitates attaching a coupler to the phone camera lens using tape and positioning the Foldscope atop the coupler. The Cellscope requires an additional bulky accessory to facilitate magnified microscopic beam paths. Meanwhile, the proposed integrated microscope is seamlessly integrated into the cell phone, enabling one-click recording and superior stability.

While the cost of modeling and nanoimprinting for the proposed integrated microscope is high before the mass production stage, typically exceeding three hundred thousand USDs and requiring the involvement of companies like Sunny Optical Technology (https://www.sunnyoptical.com/default.html), the cost for mass production of our proposed integrated microscope is less than 10 USDs, which includes expenses for plastic materials, plastic housings, IR filters, and other necessary components. The level of integration in the proposed integrated microscope is significantly higher than that of either the Cellscope or Foldscope. This leads to a much smaller and lighter design with comparable resolution, as well as an extended depth of field.

Supplementary Note 3: Comparison of the required computational sources of deep optics optimization and the proposed optimization

To apply the deep optics approach directly to the proposed integrated microscope, it is necessary to discretize the 9 surfaces in the light path (1 surface of DOE and 8 surfaces of aspherical lenses) as diffractive optical surfaces during optical propagation simulation. The sampling size dx is a critical factor in minimizing simulation artifacts and ensuring that fabrication of the optimized surfaces can yield the same outcomes as those designed.

The sampling step size dx must meet the criteria of sampling the optical propagation chirp functions in the Fourier domain, which results in a maximum sampling step size

$$dx \leq \frac{\lambda\sqrt{d_2^2 + \left(\frac{D}{2}\right)^2}}{D} \tag{1}$$

d_2 refers to the distance between the last aspherical surface and the sensor surface, which is approximately 0.7 mm in the proposed integrated microscope. The target FOV is 3.6 mm, and the aperture D is 4 mm. On the other hand, the choice of dx also needs to take into account the source field bandwidth [6]:

$$dx \leq \frac{\lambda \mathrm{d}_1}{\frac{FOV}{2} + \frac{D}{2}} \tag{2}$$

Thus the desired sampling step size dx is determined by

$$dx = \min\left(\frac{2\lambda \mathrm{d}_1}{FOV + D}, \frac{\lambda\sqrt{d_2^2 + \left(\frac{D}{2}\right)^2}}{D}\right) \tag{3}$$

According to the calculations, a sampling size of dx = 0.21 µm is desired, and would result in a matrix of 19048 × 19048 elements to describe a surface in the proposed integrated microscope system using the deep optics approach.

To optimize these surfaces, they must be expressed as tensors in deep learning frameworks like Tensorflow. In Tensorflow r1.13, the matrix must be of type Complex64, which requires 16 bytes for a single element. Storing all 9 surfaces required 4.9825×10^4 GB memory. Even when considering a rotation symmetric modeling approach, which has been shown to reduce the memory consumption and computational complexity by an order of magnitude [7], the required storage size is still around 5000 GB. To reduce the memory burden, one can calculate the optical propagation from one surface without storing other surfaces in the ram. However, this approach still needs approximately 560 GB of fresh memory for storage, not counting the size of intermediate outputs, gradients, weights of deep neural networks, and placeholders, among others. Therefore, we stated that directly applying the deep optics approach to the proposed integrated microscope would require over 600 GB of memory.

On the other hand, our proposed progressive optimization approach is memory efficient. The ray tracing step consumes less than 4 Gb of memory and can be performed on a laptop. The network optimization step only requires storing neural network-related variables such as network weights, intermediate outputs, and gradients in the memory. The unpruned network presented in the manuscript has approximately 54.88 million parameters and would require approximately 15 GB of memory, which can be easily accommodated in a single consumer GPU and is much lower than the memory requirement of the deep optics approach.

Reference

1. Song, A., et al., *Neural Anatomy and Optical Microscopy (NAOMi) Simulation for evaluating calcium imaging methods.* Journal of Neuroscience Methods, 2021(358): p. 109173.
2. Zhang, Y., et al., *Rapid detection of neurons in widefield calcium imaging datasets after training with synthetic data.* Nature Methods, 2023.
3. http://miniscope.org/index.php/Miniscope_V4.
4. Cybulski, J.S., J. Clements, and M. Prakash, *Foldscope: origami-based paper microscope.* PLoS One, 2014. **9**(6): p. e98781.
5. Skandarajah, A., et al., *Quantitative imaging with a mobile phone microscope.* PLoS One, 2014. **9**(5): p. e96906.
6. Voelz, D.G., *Computational Fourier Optics: a MATLAB tutorial*. 2011: SPIE Press.
7. Dun, X., et al., *Learned rotationally symmetric diffractive achromat for full-spectrum computational imaging.* Optica, 2020. **7**(8).
