# Supplementary material for: Large depth-of-field ultra-compact microscope by progressive optimization and deep learning
Source: Nat Commun. 2023 Jul 11;14:4118. doi: 10.1038/s41467-023-39860-0 (PMC10336131; doi:10.1038/s41467-023-39860-0)
Supplement: Supplementary file 2 — Description of additional supplementary files [file 41467_2023_39860_MOESM2_ESM.pdf]

Description of additional supplementary files

Supplementary Software 1 : Training and processing code for the proposed integrated microscope, including data generation scripts, simulation-supervision neural network training and testing scripts. See https://github.com/yuanlongo/mobilephone_EDOF for online documents.
